# ATF6 safeguards organelle homeostasis and cellular aging in human mesenchymal stem cells

**DOI:** 10.1038/s41421-017-0003-0

**Published:** 2018-01-05

**Authors:** Si Wang, Boqiang Hu, Zhichao Ding, Yujiao Dang, Jun Wu, Di Li, Xiaoling Liu, Bailong Xiao, Weiqi Zhang, Ruotong Ren, Jinghui Lei, Huifang Hu, Chang Chen, Piu Chan, Dong Li, Jing Qu, Fuchou Tang, Guang-Hui Liu

**Affiliations:** 1 National Laboratory of Biomacromolecules, CAS Center for Excellence in Biomacromolecules, Institute of Biophysics, Chinese Academy of Sciences, 100101 Beijing, China; 2 State Key Laboratory of Stem Cell and Reproductive Biology, Institute of Zoology, Chinese Academy of Sciences, 100101 Beijing, China; 3 University of Chinese Academy of Sciences, 100049 Beijing, China; 4 National Clinical Research Center for Geriatric Disorders, Xuanwu Hospital of Capital Medical University, 100053 Beijing, China; 5 Beijing Advanced Innovation Center for Genomics (ICG), College of Life Sciences, Peking University, 100871 Beijing, China; 6 Peking-Tsinghua Center for Life Sciences, Peking University, 100871 Beijing, China; 7 Gene Expression Laboratory, Salk Institute for Biological Studies, 10010 North Torrey Pines Road, La Jolla, CA 92037 USA; 8 School of Pharmaceutical Sciences, Tsinghua-Peking Joint Center for Life Sciences, IDG/McGovern Institute for Brain Research, Tsinghua University, 100084 Beijing, China; 9 Ministry of Education Key Laboratory of Cell Proliferation and Differentiation, 100871 Beijing, China; 10 Biomedical Institute for Pioneering Investigation via Convergence, Peking University, 100871 Beijing, China; 11 Key Laboratory of Regenerative Medicine of Ministry of Education, Institute of Aging and Regenerative Medicine, Jinan University, 510632 Guangzhou, China

## Abstract

Loss of organelle homeostasis is a hallmark of aging. However, it remains elusive how this occurs at gene expression level. Here, we report that human mesenchymal stem cell (hMSC) aging is associated with dysfunction of double-membrane organelles and downregulation of transcription factor ATF6. CRISPR/Cas9-mediated inactivation of *ATF6* in hMSCs, not in human embryonic stem cells and human adipocytes, results in premature cellular aging, characteristic of loss of endomembrane homeostasis. Transcriptomic analyses uncover cell type-specific constitutive and stress-induced ATF6-regulated genes implicated in various layers of organelles’ homeostasis regulation. *FOS* was characterized as a constitutive ATF6 responsive gene, downregulation of which contributes to hMSC aging. Our study unravels the first ATF6-regulated gene expression network related to homeostatic regulation of membrane organelles, and provides novel mechanistic insights into aging-associated attrition of human stem cells.

## Introduction

The cellular proteome is tightly regulated by the proteostasis network, a complex system that controls protein synthesis, folding, and degradation^1–3^. Preserving the stability and functionality of proteomes is essential for the proper cellular function and biological process. Loss of proteostasis is considered as one of the hallmarks of aging^4–9^. More evidence shows that accumulation of misfolded or unfolded proteins contributes to the development of aging-related diseases^1, 4, 10^. Endoplasmic reticulum (ER) is the largest intracellular endomembrane system, enabling protein quality control, Ca^2+^ ion homeostasis, and organelle communication^11^. ER executes the protein quality control via two pathways. One is mediated by ER-resident molecular chaperones and enzymes to ensure proper protein folding. The other is ER-associated degradation (ERAD) pathway^2^, by which unfolded or misfolded proteins in the ER are transported to the cytoplasm for degradation through ubiquitin proteasome system^1–3^.

In addition, ER is connected with other membrane-bound organelles. ER not only physically connects with the outer nuclear membrane and communicates with Golgi apparatus by vesicle transport, but also contacts with mitochondria for coupling mtDNA synthesis and contributes to biogenesis of autophagosomes by cross-talking with mitochondria^12–14^. Indeed, loss of the architectural and functional integrity of these membrane organelles has been reported for aging and several age-associated disorders^15, 16^. For instance, senescent cells frequently show alterations in nuclear envelope (NE), mitochondria, ER, and Golgi^15–18^. The molecular mechanisms underpinning these changes, however, remain unexplored.

ER stress is sensed by ER transmembrane proteins, including activating transcription factor 6 (ATF6), which initiate a series of ER-to-nucleus signaling cascades to protect against cytotoxicity of accumulated unfolded or misfolded proteins and restore the ER homeostasis^19–21^. Upon ER stress, the membrane-bound ATF6 traffics from the ER to the Golgi apparatus where it is processed to active form by sequential cleavage^19, 22^. The cleaved fragment is subsequently released from the Golgi membrane and functions as nuclear transcription factor, which regulates the transcription of a number of unfolded protein response (UPR) genes^23–26^. ATF6 normally binds to the bipartite ER stress response element (ERSE) I (CCAAT-N9-CCACG/A), or ERSE II (ATTGG-N1-CCACG) of the promoter of target genes, in the presence of the CCAAT box binding factors^20^. So far, it is still unclear whether ATF6 plays any role in regulating human cellular homeostasis and aging.

In this study, by combining human stem cell-directed differentiation and gene editing techniques, we investigated the effect of ATF6 absence in three types of human cells (human embryonic stem cells (hESCs), human mesenchymal stem cells (hMSCs), and human white adipocytes (hWAPCs)), and identified ATF6 as a master regulator of hMSC homeostasis. Inactivation of ATF6 in hMSCs led to multiple organelles’ dysfunction and accelerated cellular senescence, a process in which FOS functioned as one of the mediators.

## Results

### Accelerated functional decay in ATF6-deficient hMSCs

**Fig. 1:**
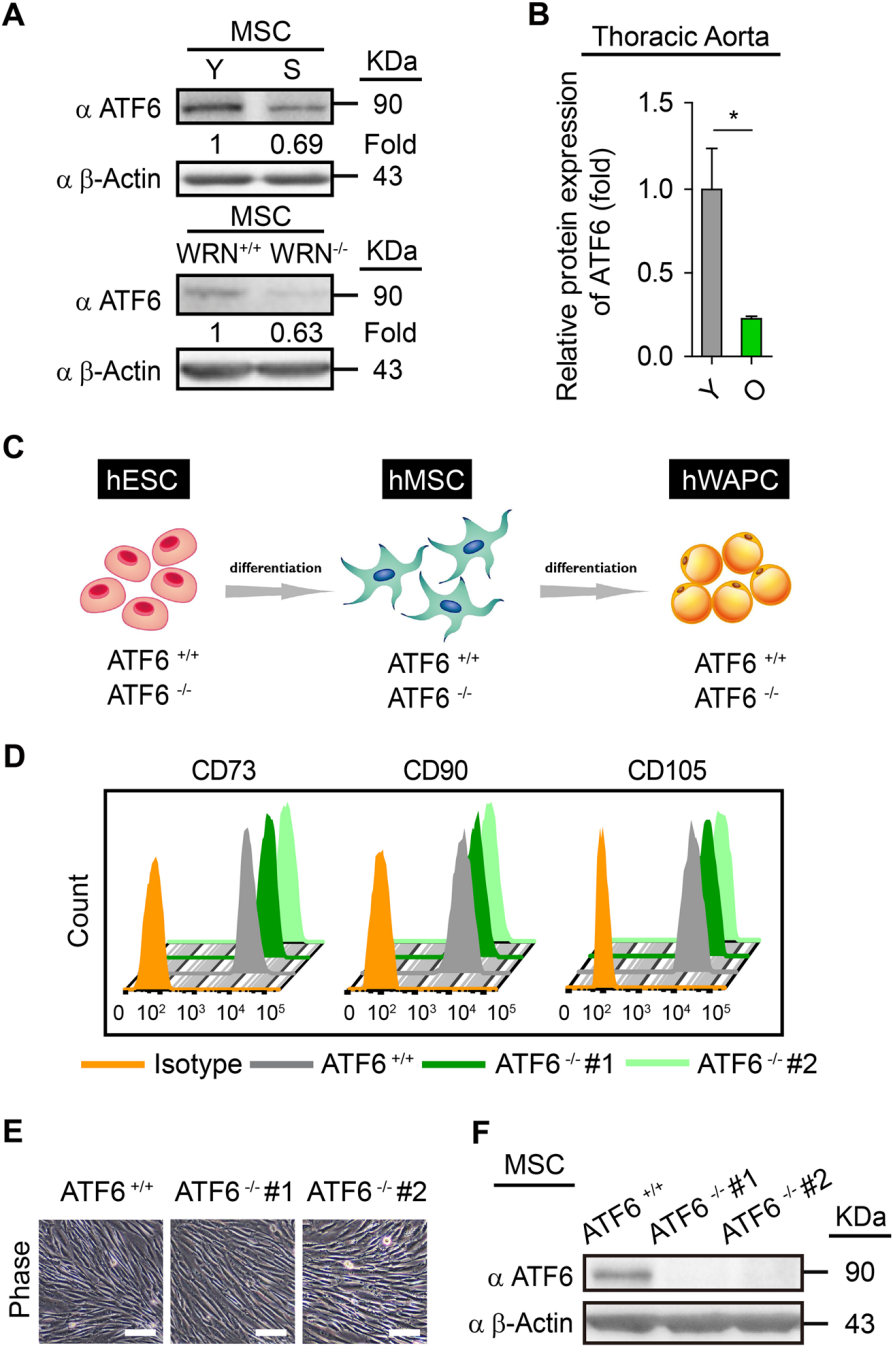
Generation and characterization of ATF6-deficient hMSCs. **a** Western blotting showing decreased expression of ATF6 in replicative senescent and Werner Syndrome (WS) hMSC. β-Actin was used as the loading control. Y young, S senescent. **b** Decreased expression of ATF6 was observed in thoracic aortas from aged mice. Thoracic aortas from three young (6-week-old) and three old (15-month-old) mice were collected and then subjected to western blotting. The protein levels of ATF6 and GAPDH (loading control) were quantified by densitometry with Image J software. Data were presented as mean ± SD, *n* = 3, *P < 0.05, Y young, O old. **c** Schematic representation of differentiation of WT (*ATF6*^*+/+*^*)* and ATF6-deficient (*ATF6*^−/−^*)* hESCs into hMSCs, then into hWAPCs. **d** FACS analysis indicated the expression of cell surface markers CD73, CD90, and CD105 in WT and ATF6-deficient hMSCs. *ATF6*^−/−^ #1 and *ATF6*^−/−^ #2 were two independent *ATF6*^−/−^ hESC lines obtained using CRISPR/Cas9-mediated gene targeting technique with sgRNA1 and sgRNA2, respectively. **e** Representative bright-field images showing the morphology of WT and ATF6-deficient hMSCs differentiated from WT and ATF6-deficient hESCs, respectively. Scale bar, 50 μm. **f** Western blotting showing the protein level of ATF6 in WT and ATF6-deficient hMSCs. β-Actin was used as the loading control. See also Supplementary Figure S1 and S2

To explore the relationship between protein quality control and human stem cell aging, we checked the expression of a series of UPR proteins in replicative senescent hMSCs and premature aging (Werner Syndrome, WRN-deficient) hMSCs^27–30^ (Supplementary Figure S1A). Western blotting demonstrated that the expression of the ATF6 protein was diminished in aged hMSCs (Fig. 1a). Moreover, reduced ATF6 expression was observed during aging in mouse thoracic aorta (Fig. 1b, Supplementary Figure S1B), where MSCs constitute a major component of tunica adventitia^29, 31^. We did not observe senescence-associated downregulation of other UPR genes (Supplementary Figure S1A).

To investigate the potential role of ATF6 downregulation in hMSC aging, we first generated ATF6-deficient hESCs using CRISPR/Cas9-mediated homologous recombination approach (Supplementary Figure S1C, D). Loss of mRNA and protein expression of ATF6 in ATF6-deficient hESCs (*ATF6*^−/−^ hESCs) was verified by quantitative reverse transcription-PCR (qRT-PCR) and western blotting, respectively (Supplementary Figure S2A, B). Like wild-type (WT) hESCs, ATF6-null hESCs expressed pluripotency markers including *NANOG*,* OCT4*, and *SOX2* (Supplementary Figure S2C, D) and displayed DNA-hypomethylated *OCT4* promoter (Supplementary Figure S2E). In addition, *ATF6*^−/−^ hESCs maintained the in vivo differentiation potential toward three germlayer lineages, which were demonstrated by teratoma formation assay (Supplementary Figure S2F). *ATF6*^−/−^ hESCs were cultured for more than 50 passages without losing normal ESC-like cellular morphology, pluripotency markers, growth kinetics, as well as normal karyotype (Supplementary Figure S2G–I).

**Fig. 2:**
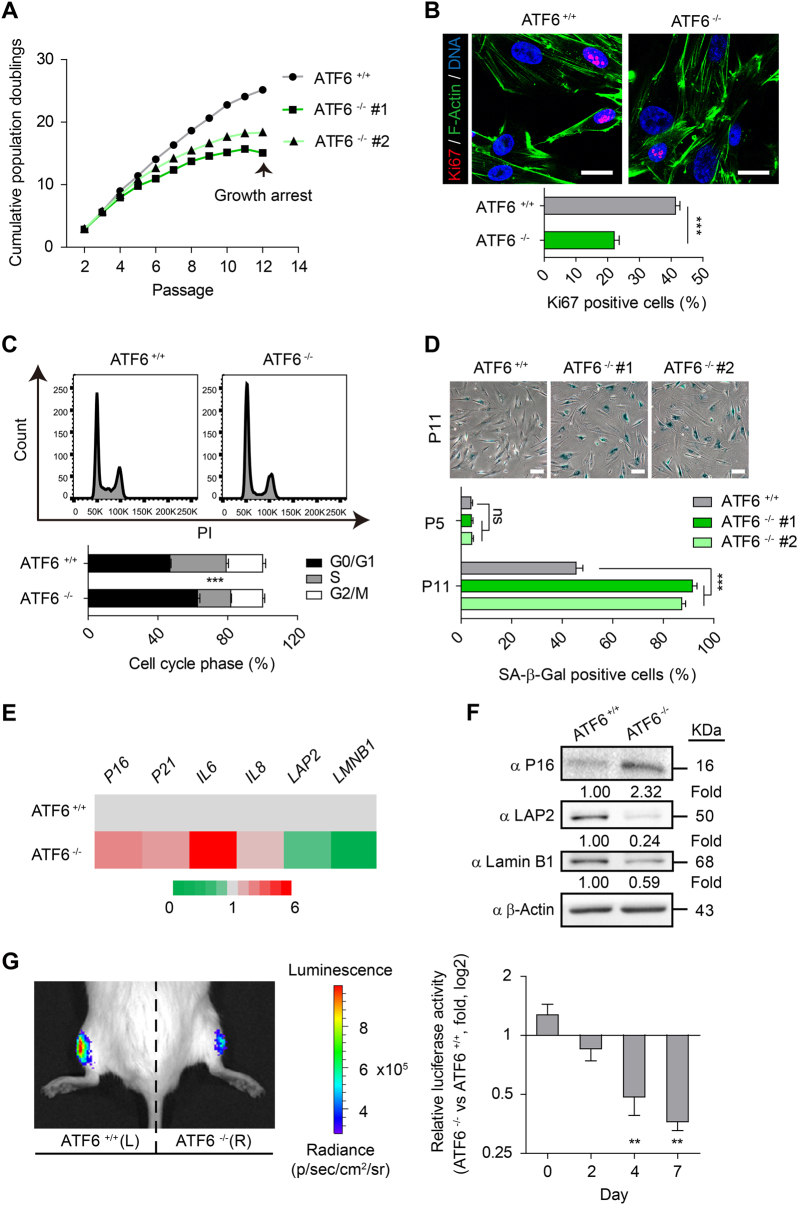
ATF6-deficient hMSCs exhibit phenotypes associated with premature cellular senescence. **a** Growth curve showing the cumulative population doublings of WT and ATF6-deficient hMSCs over passages. **b** Immunostaining of Ki67 showing decreased cell proliferation of ATF6-deficient hMSCs compared to WT control; the percentages of Ki67 positive cells were shown in the bottom panel. Scale bar, 20 μm. Data were presented as the mean ± SEM, *n* = 3, ***P < 0.001. **c** Cell cycle profiles showing the decreased S-phase cells in ATF6-deficient hMSCs compared to WT control. Data were presented as the mean ± SEM, *n* = 3, ***P < 0.001. **d** SA-β-Gal staining of WT and ATF6-deficient hMSCs at passages 5 and 11, respectively. The percentages of SA-β-Gal-positive cells are shown in the bottom panel. Data were presented as mean ± SEM, *n* = 3, ns not significant, ***P < 0.001. Scale bar, 20 μm. **e** The heatmap showing the qRT-PCR analysis of the expression of senescence marker genes in WT and ATF6-deficient P10 hMSCs. Transcript levels were normalized to the WT control. **f** Western blotting analysis of P16, LAP2 and Lamin B1 in WT and ATF6-deficient hMSCs. β-Actin was used as the loading control. **g** Analysis of luciferase activity by imaging system (IVIS) showing premature attrition of ATF6-deficient hMSCs *in vivo*. WT (1 × 10^6^, left) and ATF6-deficient (1 × 10^6^, right) hMSCs (Passage 10) infected with luciferase lentivirus were transplanted into the tibialis anterior (TA) muscles of four immunodeficient mice. Luciferase activities were imaged and quantified at days 0, 2, 4, and 7 after transplantation. Data were presented as the ratios of *ATF6*^−/−^ to *ATF6*^+/+^ (fold), mean ± SD, *n* = 4, **P < 0.01. See also Supplementary Figure S3

We next differentiated ATF6-deficient and WT hESCs into hMSCs to investigate whether ATF6 deficiency could result in accelerated attrition of hMSC pool (Fig. 1c)^27, 29, 30, 32–35^. Both ATF6-deficient hMSCs (*ATF6*^−/−^ hMSCs) and WT hMSCs were positive for mesenchymal progenitor markers, including CD73, CD90, and CD105 (Fig. 1d, e), and negative for non-MSC markers such as CD34, CD43, and CD45 (Supplementary Figure S3A). We verified the absence of mRNA and protein expression of ATF6 in *ATF6*^−/−^ hMSCs (Supplementary Figure S3B, Fig. 1f). To test whether transcription activity of ATF6 was abolished in the ATF6-deficient hMSCs, we examined the expression changes of BIP, HERPUD1, and ERO1B, known ATF6 target genes^36^. As expected, reduced induction of BIP, HERPUD1, and ERO1B was observed in the ATF6-deficient hMSCs when treated with ER stress inducer tunicamycin (TM)^19^ in comparison with WT cells (Supplementary Figure S3C–E). We next compared WT and *ATF6*^−/−^ hMSCs in multipotent differentiation potential. While both WT and *ATF6*^−/−^ hMSCs differentiated into human white adipocytes (hWAPCs) with comparable efficiency (Supplementary Figure S3F), *ATF6*^−/−^ hMSCs exhibited impaired differentiation abilities toward chondrocytes and osteoblasts (Supplementary Figure S3F–H). Next we investigated whether ATF6 is required for maintaining the long-term cellular homeostasis of hMSCs. As shown in Fig. 2a–d, *ATF6*^−/−^ hMSCs displayed features characteristic of premature senescence, including progressive impairment of cell proliferation ability (Fig. 2a, Supplementary Figure S3I), reduced Ki67 positive cells (Fig. 2b), decreased percentage of S-phase cells (Fig. 2c), and increased senescence-associated (SA)-β-Gal activity (Fig. 2d). In addition, expression levels of senescence markers including P16, P21, IL-6, and IL-8 were upregulated, while Lamin B1 and LAP2 were downregulated in *ATF6*^−/−^ hMSCs relative to WT hMSCs (Fig. 2e, f). Next, we investigated whether *ATF6*^−/−^ hMSCs underwent accelerated attrition in vivo by implanting WT and *ATF6*^−/−^ hMSCs expressing luciferase into the tibialis anterior (TA) muscle of immunodeficient mice^27, 29, 34^. *ATF6*^−/−^ hMSCs exhibited accelerated in vivo decay when compared to WT hMSCs (Fig. 2g). To confirm whether loss of ATF6 contributes to above phenotypes, *ATF6*^−/−^ hMSCs were transduced with lentiviral vector encoding a constitutively active version of ATF6 (ATF6-CA)^19, 23, 26^. Overexpression of ATF6-CA partially restored the cellular proliferation ability of *ATF6*^−/−^ hMSCs (Supplementary Figure S3J–M). Together, these results identify ATF6 as a geroprotector for hMSCs.

### Disruption of membrane-bound organelles in ATF6-deficient hMSCs

**Fig. 3:**
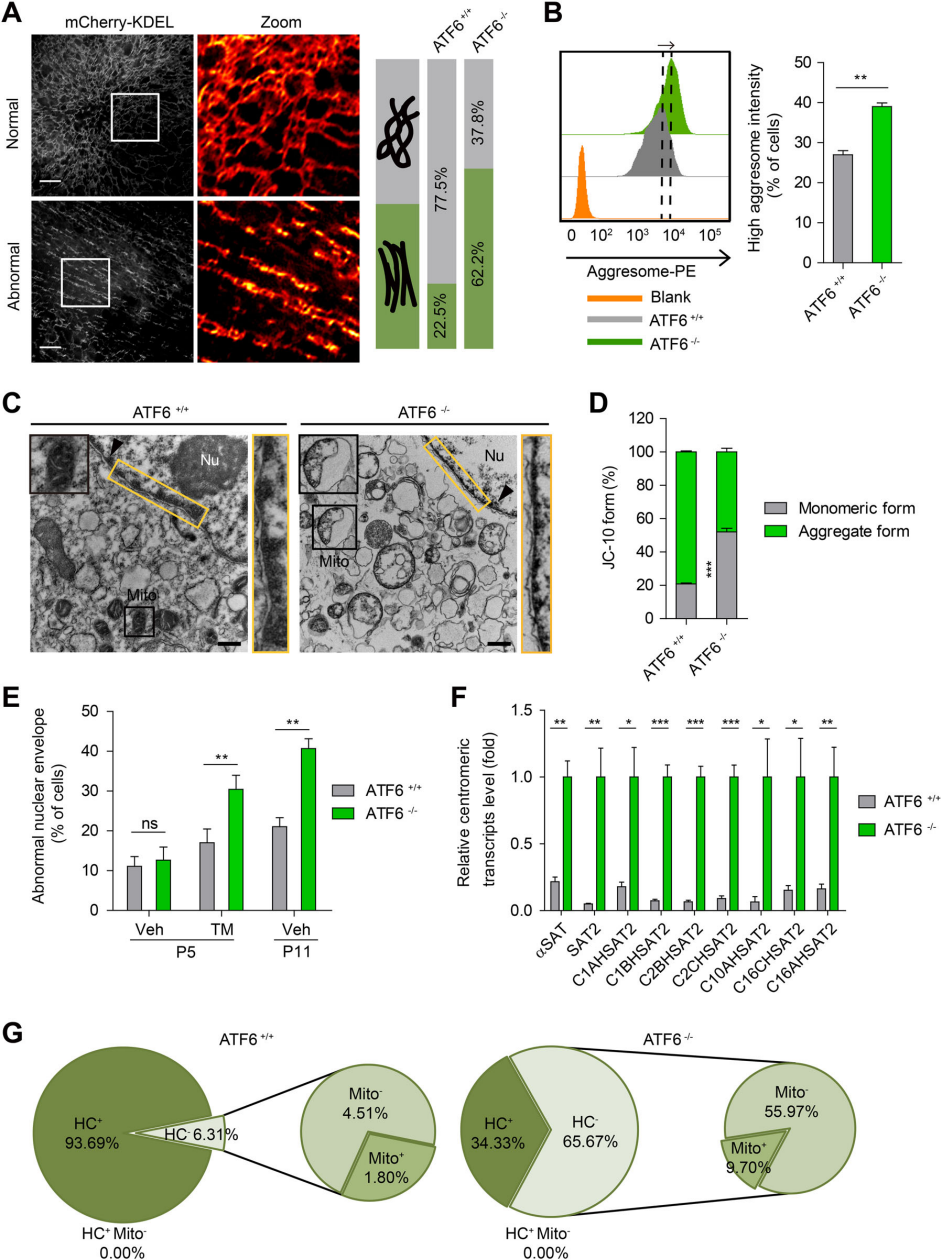
ATF6 is required for homeostasis of ER and ER-associated membrane organelles in hMSCs. **a** Representative 2D-SIM images showing the ER morphology of WT and ATF6-deficient hMSCs. hMSCs were transfected with KDEL-mCherry and imaged with TIRF-SIM system. Forty-nine WT and 45 ATF6-deficient hMSCs were imaged, respectively. The percentages of cells with normal/abnormal ER structure is shown in the right panel. Scale bar, 3 μm. **b** The aggresomes were labeled with aggresome probe, and the aggresome intensity of WT and ATF6-deficient hMSCs was analyzed by FACS. Data were presented as the mean ± SEM, *n* = 3, **P < 0.01. **c** Representative TEM images showing reduced heterochromatin and increased abnormal mitochondria in the ATF6-deficient hMSCs. Scale bar, 500 nm. Nu nucleus, Mito mitochondrion. Arrow head denotes the heterochromatin. **d** The mitochondria dysfunction in ATF6-deficient hMSCs was demonstrated by increased percentage of cells with monomeric form of JC-10 by flow cytometry analysis. Data were presented as the mean ± SEM, *n* = 3, ***P < 0.001. **e** The percentages of cells with abnormal NE morphology was calculated based on immunostaining of Lamin A/C protein. Data were presented as the mean ± SEM, *n* = 3, ns not significant, **P < 0.01. **f** qRT-PCR analysis of centromeric repetitive element transcripts in WT and ATF6-deficient hMSCs. Data were presented as the mean ± SEM, *n* = 3, *P < 0.05, **P < 0.01, ***P < 0.001. **g** Pie charts showing the distribution of cells with reduced heterochromatin and/or abnormal mitochondria observed by TEM in WT and ATF6-deficient hMSCs. “HC” denotes heterochromatin, “HC^−^” denotes reduced heterochromatin, “Mito” denotes mitochondria, “Mito^−^” denotes abnormal mitochondria. One-hundred and eleven WT hMSCs and 134 ATF6-deficient hMSCs were imaged and calculated, respectively. See also Supplementary Figure S4

Since ATF6 is a transcription factor accounting for transcriptional activation of ER-related genes^36, 37^, we first examined whether ATF6 deficiency results in architectural and functional changes in the ER. Structured-illumination microscopy (SIM) with high resolution demonstrated that ER in *ATF6*^−/−^ hMSCs displayed impaired ER tubular network. In comparison with WT hMSCs, the percentage of cells with unbranched ER tubules increased upon ATF6 depletion^38, 39^ (Fig. 3a, Supplementary Movie 1 and 2). We observed accumulated protein aggresomes in ATF6-deficient hMSCs, supporting an impairment of ER-regulated proteostasis (Fig. 3b)^5^. In addition, ER Ca^2+^ storage was reduced in *ATF6*^−/−^ hMSCs (Supplementary Figure S4A). These observations support the notion that ATF6 safeguards the architectural and functional integrity of ER in hMSCs.

As ER is in physical contact with mitochondria^13, 14, 40^, we hypothesize that ATF6 deficiency can result in altered mitochondria morphology and function. Transmission electron microscopy (TEM) revealed more abnormal mitochondria morphologies, e.g., swollen, hollow, and circled, progressively appeared in *ATF6*^−/−^ hMSCs upon serial passaging (Fig. 3c). In addition, a greater number of *ATF6*^−/−^ hMSCs lost the mitochondrial membrane potential (MMP) compared to WT hMSCs (Fig. 3d).

Additionally, since NE also connects ER membrane, we next investigated whether NE and its associated nuclear lamina proteins are affected by ATF6 deficiency. Immunostaining with anti-Lamin A/C antibody indicated that the NE became abnormally shaped in *ATF6*^−/−^ hMSCs upon serial passaging (Fig. 3e, Supplementary Figure S4B). Up to 40% misshapen nuclei were observed in late passage *ATF6*^−/−^ hMSCs (relative to 20% in WT hMSCs). In addition, *ATF6*^−/−^ hMSCs frequently exhibited loss of inner nuclear transmembrane protein LAP2 (a protein tethering heterochromatin to NE), as well as reduced heterochromatin structure underneath the NE (Fig. 3c, g, Supplementary Figure S4C), all of which are known marks of senescent hMSCs^27, 30, 32^. Moreover, in line with heterochromatin destabilization, levels of centromeric repetitive sequence transcripts were upregulated in ATF6-deficient hMSCs (Fig. 3f). These architectural changes in NE seem to be linked to ER dysfunction, as ER protein Calreticulin and Lamins were frequently co-localized along the distorted NE in *ATF6*^−/−^ hMSCs (Supplementary Figure S4D). A more direct link between ER stress and NE abnormality came from the observation that early passage *ATF6*^−/−^ hMSCs, when treated with TM, displayed severe NE distortion as observed in late passage *ATF6*^−/−^ hMSCs (Fig. 3e). Furthermore, we observed that mitochondrial alterations were coupled with NE abnormalities and reduced heterochromatin in ATF6-deficient hMSCs (Fig. 3g). These results indicate that absence of ATF6 in hMSCs results in coordinated architectural and functional alterations of membrane organelles.

### Gene expression changes in ATF6-deficient hMSCs

As a transcription factor, ATF6 exerts biological activities primarily via transcriptional activation of its target genes^36, 37, 41^. To investigate whether the gene transcription programs were impaired in *ATF6*^−/−^ hMSCs, we performed genome-wide RNA sequencing (RNA-seq) analysis using WT and *ATF6*^−/−^ hMSCs. As controls, WT and ATF6-depleted hESCs, as well as their hWAPCs counterparts, were subjected to RNA sequencing (Supplementary Figure S5A, B).

**Fig. 4:**
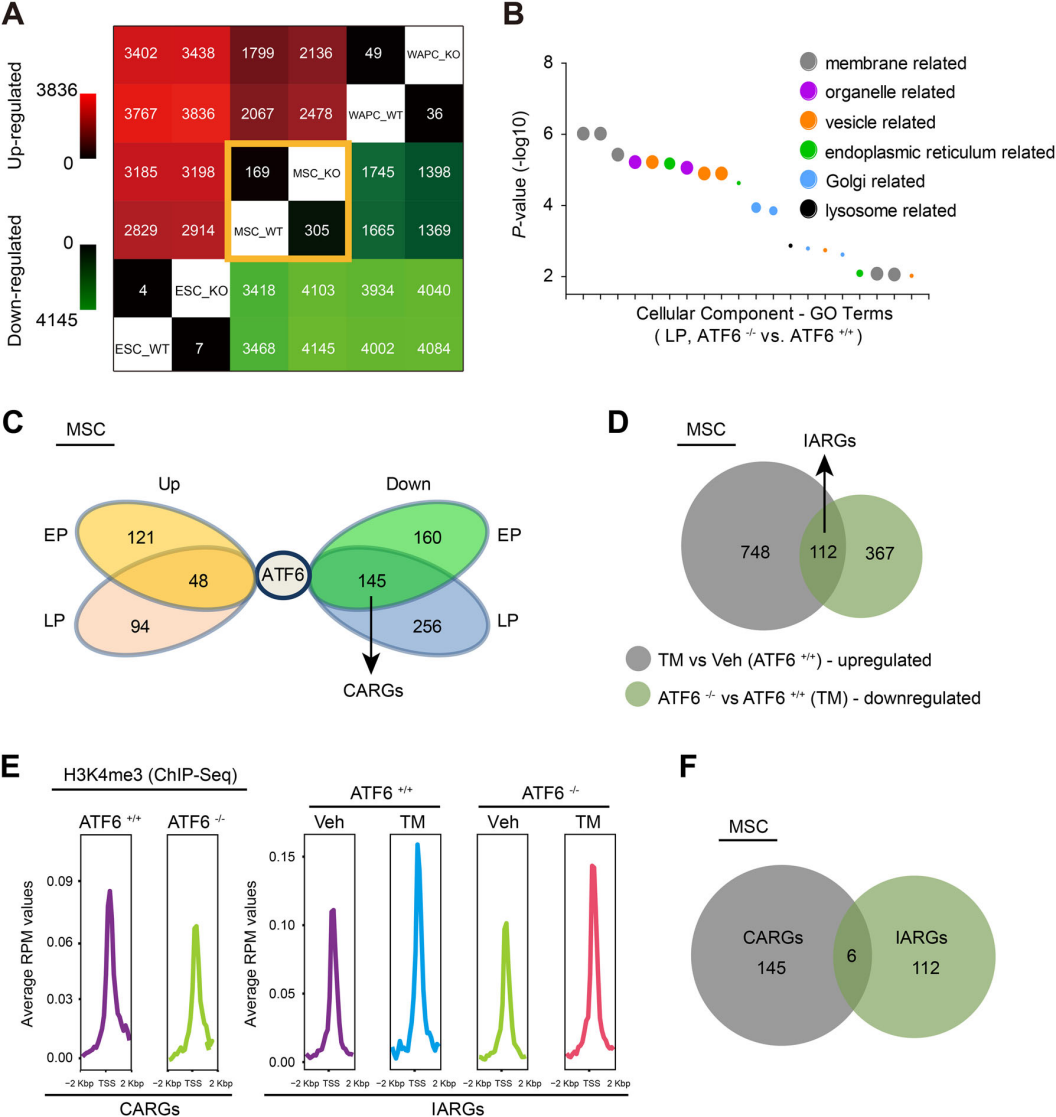
Gene expression analysis of WT and ATF6-deficient hMSCs. **a** Heatmap showing the number of differentially expressed genes (DEG) in WT and ATF6-deficient hESC, hMSC and hWAPC. WT stands for wild-type, KO stands for knockout of *ATF6*. **b** Gene ontology (GO) analysis (cellular component) of the significantly downregulated genes in the late passage hMSCs upon ATF6 deletion. **c** A venn diagram showing that the early passage (EP early passage, P5) and late passage (LP late passage, P10) hMSCs share significantly up- or downregulated genes in ATF6-deficient hMSCs relative to WT hMSCs. CARGs stands for constitutive ATF6 responsive genes. **d** A venn diagram showing 112 IARGs as defined by common genes between upregulated in WT hMSC after TM treatment (gray) and downregulated in ATF6-deficient vs. WT hMSCs upon TM treatment (green). IARGs stands for induced ATF6 responsive genes. **e** Average H3K4me3 signals at the gene promoters of CARGs and IARGs in WT and ATF6-deficient hMSCs treated with either vehicle or TM. **f** A venn diagram showing six genes shared by CARGs and IARGs. See also Supplementary Figure S5–S11

We observed 169 upregulated and 305 downregulated genes in early passage *ATF6*^−/−^ hMSCs in comparison with WT hMSCs (Fig. 4a, Supplementary Table S2). In contrast, there were only four upregulated and seven downregulated genes in *ATF6*^−/−^ hESCs, and 49 upregulated and 36 downregulated genes in *ATF6*^−/−^ hWAPCs compared with WT cells, respectively (Fig. 4a, Supplementary Table S2). These results were in line with the absence of discernible phenotypes in *ATF6*^−/−^ hESCs (Supplementary Figure S2G, H), suggesting that hMSCs are more susceptible to *ATF6* deficiency than the other two cell types. Within the 305 genes downregulated in *ATF6*^−/−^ hMSCs, GO term analysis revealed that 14 genes are associated with bone development (Supplementary Figure S6A, C, Supplementary Table S5), supporting the observations that *ATF6*^−/−^ hMSCs were refractory to osteogenesis and chondrogenesis (Supplementary Figure S3F–H). In addition, we identified a panel of downregulated genes related to cellular proliferation (Supplementary Figure S6D, Supplementary Table S5), consistent with accelerated aging phenotypes observed in ATF6-deficient MSCs.

The differentially expressed genes between WT and *ATF6*^−/−^ hMSCs at late passage were primarily clustered to architectural proteins of membrane organelle (Fig. 4b, Supplementary Figure S7A, Supplementary Table S3, S6). There were 48 upregulated and 145 downregulated genes in ATF6-deficient MSCs both in early and late passages when compared with WT hMSCs (Fig. 4c). We focused on the 145 downregulated genes, which were hereafter referred as to “constitutive ATF6 responsive genes (CARGs)” (Fig. 4c, Supplementary Table S4). Protein function analysis indicated that most of these genes encode membrane/transmembrane proteins, which constitute major membrane components of ER, Golgi, mitochondria, and other organelles. Several CARGs were associated with the GO term of “calcium signaling” (Supplementary Figure S6B), in agreement with the observed impaired calcium homeostasis in *ATF6*^−/−^ hMSCs. Chromatin immunoprecipitation (ChIP) sequencing (ChIP-seq) demonstrated an enrichment of transcriptionally active H3K4me3 marks at the promoters of CARGs in WT hMSCs, but either reduced or absent in ATF6-deficient hMSCs (Fig. 4e, Supplementary Figure S8A, B). These changes in gene expression underlie the cellular defects in *ATF6*^−/−^ hMSCs, further supporting a role of ATF6 in regulating organelle homeostasis and hMSC aging.

### Identification of ER stress-induced ATF6-responsive genes in hMSCs

To examine ATF6’s role in hMSCs in the presence of ER stress, WT and *ATF6*^−/−^ hMSCs, hESCs, and hWAPCs were treated with TM for 12 h, and global gene expression profiles were analyzed by RNA-seq (Supplementary Figure S7B, S9A, B). TM treatment resulted in 550, 860, and 1006 upregulated genes in WT hMSCs, hESCs, and hWAPCs, respectively (Supplementary Figure S9A). The majority of these genes related to the GO term “ER stress” (Supplementary Figure S7C, Supplementary Table S7), indicating the successful induction of ER stress responses in all three cell types. Next, we focused on common genes between upregulated by TM treatment in WT cells and downregulated in *ATF6*^−/−^ vs. *ATF6*^+/+^ cells after TM treatments (Fig. 4d, Supplementary Figure S10A). We named these genes as “induced ATF6 responsive genes (IARGs)” (Fig. 4d, Supplementary Table S4); 112, 12, and 48 IARGs were identified in hMSCs, hESCs, and hWAPCs, respectively (Fig. 4d, Supplementary Figure S10A, C). Among them, 10 overlapping genes were identified among hMSCs, hESCs, and hWAPCs, and 84 IARGs were specific for hMSCs (Supplementary Figure S10B). The expression patterns of IARGs in hMSCs were consistent with the levels of transcriptionally active H3K4me3 mark in their gene promoters (Fig. 4e, Supplementary Figure S8A, C). In MSCs, only six IARGs were overlapped with CARGs (Fig. 4f), suggesting that ATF6 mediates transcriptional activation of different sets of genes in basal and ER stress conditions, respectively. Finally, GO term and gene–gene interaction network analyses demonstrated that hMSC-specific IARGs were enriched for terms related to processes such as “response to endoplasmic reticulum stress” (*P* = 2.26E−23), “endoplasmic reticulum unfolded protein response” (*P* = 8.06E-17), “ER-associated ubiquitin-dependent protein catabolic process” (*P* = 6.37E−12), “protein folding” (*P* = 4.01E−11), etc. (Supplementary Figure S10D, Supplementary Figure S11, Supplementary Table S8), which is consistent with the known function of ATF6 in regulating UPR.

### Characterization of CARGs and IARGs

**Fig. 5:**
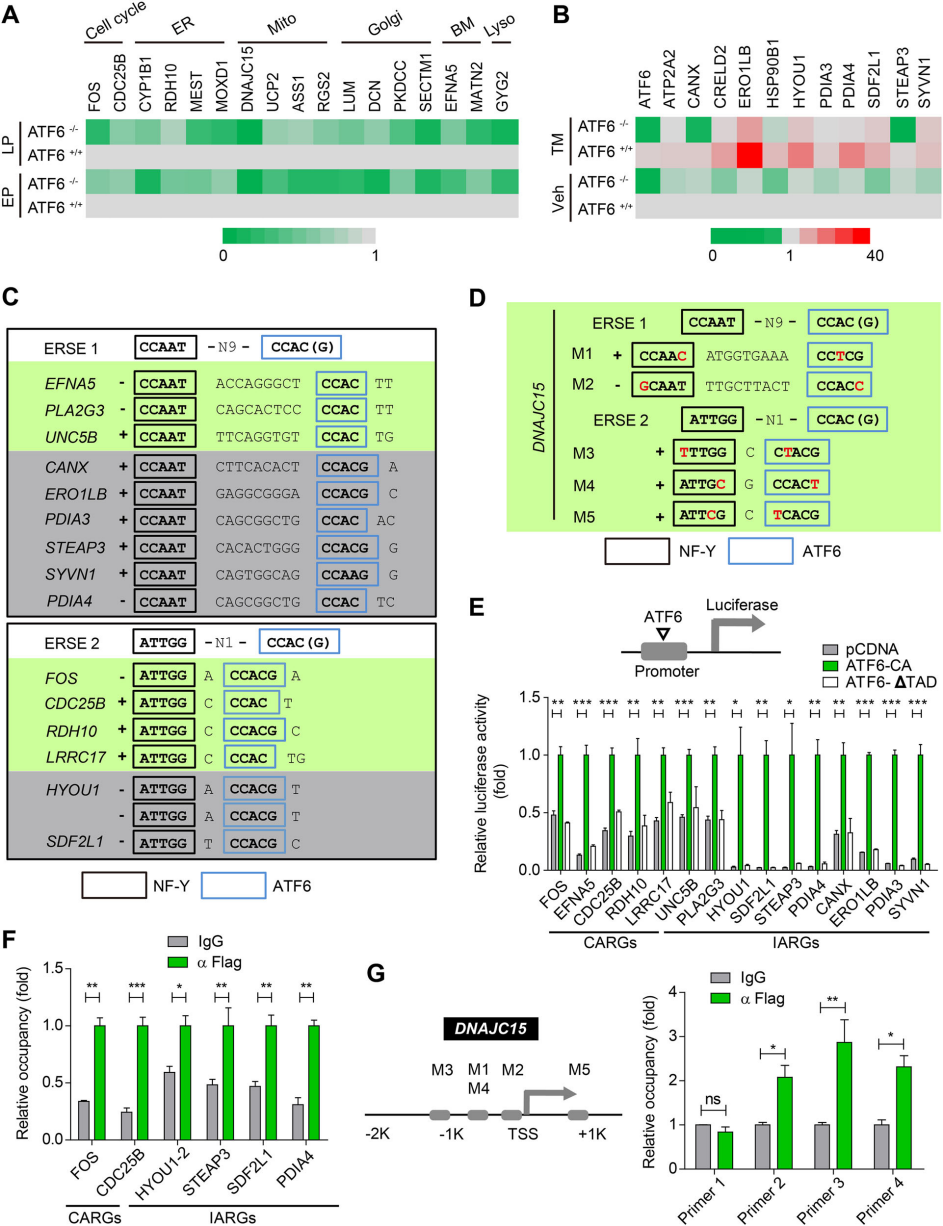
Characterization of a panel of ATF6-regulated genes. **a** The heatmap showing gene expression changes of CARGs in EP (Passage 5) and LP (Passage 10) of WT and ATF6-deficient hMSCs. **b** The heatmap showing gene expression changes of IARGs in vehicle and TM-treated WT and ATF6-deficient hMSCs. **c** The motif pattern analysis in the promoter region showing that the canonical ERSE I and ERSE II are present in the promoters of several CARGs and IARGs. The promoter sequences spanning of upstream 10 kb and downstream 2 kb of TSS were examined. Consensus sequence is shown in the indicated boxes. Black box is the consensus sequence to which NF-Y binds, blue box is the consensus sequence to which ATF6 binds. **d** Promoter analysis showing that the non-canonical ERSE I and ERSE II are also present in the promoters of *DNAJC15*. M motif. **e** Luciferase reporter assay showing that the putative promoters containing the ERSE I/ERSE II of several CARGs and IARGs could be activated by ATF6-CA, but not ATF6-ΔTAD mutants. CA constitutively active; ΔTAD transactivation domain-deleted. Data were presented as mean ± SEM, *n* = 3, *P < 0.05, **P < 0.01, ***P < 0.001. **f** ChIP-PCR analysis showing the binding of Flag-ATF6 to several promoters of CARGs and IARGs. The promoter was amplified by PCR from either genomic DNA as input or anti-Flag immunoprecipitated DNA. Data were presented as mean ± SEM, *n* = 3, *P < 0.05, **P < 0.01, ***P < 0.001. **g** ChIP-PCR showing the binding of Flag-ATF6 with non-canonical ERSE I and ERSE II motifs in the promoter of *DNAJC15*. Primer pairs 1, 2, 3, and 4 were used to amplify the immunoprecipitated DNA spanning the putative motif 3, motif 1/4, motif 2, and motif 5, respectively. M motif. Data were presented as the mean ± SEM, *n* = 3, ns not significant, *P < 0.05, **P < 0.01

Among the 145 CARGs and 112 IARGs in hMSCs, an array of known ATF6-regulated genes were identified, including BIP, HERPUD1, CALR, DERL3, etc.^36^; however, most of these genes were new and not characterized previously. We next verified the expression of several selected genes in response to replicative stress or ER stressor in WT and ATF6-deficient hMSCs using qRT-PCR (Fig. 5a, b, Supplementary Figure S12A). To further test whether these genes are ATF6 targets, we scanned their promoter regions spanning from −10,000 bp to 2000 bp of the transcription start site (TSS) for potential ERSE. We found that the promoters of 21 genes contained classic ERSE, including *FOS*, *CDC25B*, *EFNA5*, *STEAP3*, *SDF2L1*, etc., which have not been reported as ATF6 targets previously (Fig. 5c). Apart from the canonical ERSE, we also identified some non-typical sequences resembling ERSE in the promoters of several genes, including a mitochondrial gene *DNAJC15* (Fig. 5d).

To know whether CARGs and IARGs are directly activated by ATF6, we cloned the promoters of several CARGs and IARGs upstream of the luciferase reporter, followed by transfection into HEK293T cells together with an expression vector encoding a constitutively active ATF6 (ATF6-CA) or transactivation domain-deleted ATF6 (ATF6-ΔTAD). We found that the promoters of these genes, including *FOS*, *CDC25B*, *EFNA5*, *STEAP3*, *SDF2L1*, and etc., were activated by ATF6-CA but not ATF6-ΔTAD (Fig. 5e). In addition, the putative ERSE-contained sequence of *FOS* functioned as an enhancer which was transactivated by ATF6-CA (Supplementary Figure S12B). To know whether ATF6 is able to bind directly to the promoters of these candidate genes, we performed ChIP-qPCR in *ATF6*^−/−^ hMSCs reconstructed with Flag-ATF6-CA, using anti-Flag antibody. The results demonstrated that ATF6 was enriched at endogenous promoters of several CARGs and IARGs, including *FOS*, *CDC25B*, *PDIA4*, *SDF2L1*, and *STEAP3* (Fig. 5f). We also found the binding of ATF6 to the non-canonical ERSE in three regions of *DNAJC15* promoter (Fig. 5g). Together, these results characterized a panel of novel ATF6-responsive genes in hMSCs.

### Downregulation of *FOS* partially accounts for stem cell attrition in ATF6-deficient hMSCs

**Fig. 6:**
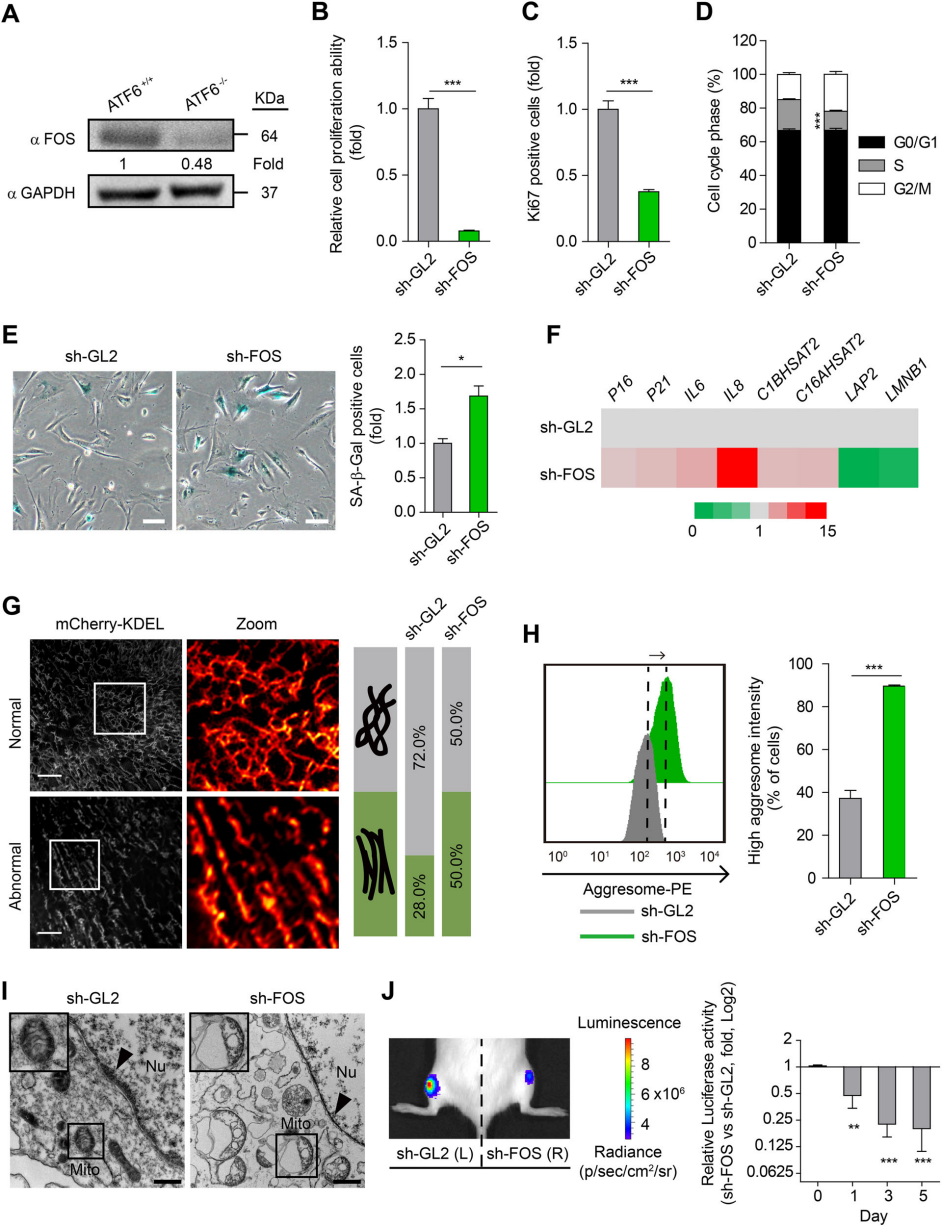
Knockdown of *FOS* partially mimics the cellular senescent phenotypes of ATF6-deficinet hMSCs. **a** Western blotting showing reduced protein level of *FOS* in *ATF6*^−/−^ hMSCs. GAPDH was used as the loading control. **b** Decreased cell proliferation ability after knockdown of *FOS* in WT hMSCs. Data were presented as mean ± SEM, *n* = 3, ***P < 0.001. **c** Decreased Ki67 positive cells in the *FOS*-knockdown hMSCs. Data were presented as mean ± SEM, *n* = 3, ***P < 0.001. **d** Cell cycle analysis showing decreased S-phase cells after knockdown of *FOS*. Data were presented as mean ± SEM, *n* = 3, ***P < 0.001. **e** Representative images showing knockdown of *FOS* led to increased SA-β-Gal staining positive cells (left). The fold  of positive cells are shown in the right panel. Data were presented as the mean ± SEM, *n* = 3, *P < 0.05. Scale bar, 50 μm. **f** The heatmap showing knockdown of *FOS* led to mRNA expression changes of senescent marker genes. **g** ER imaging showing increased impaired ER in *FOS*-knockdown hMSCs. Fifty WT and 46 ATF6-deficient hMSCs were imaged, respectively, and the percentages of cells with normal/abnormal ER structure are shown in the right panel. Scale bar, 3 μm. **h** Increased aggresome intensity after knockdown of *FOS* indicated by FACS analysis. Data were presented as mean ± SEM, *n* = 3, ***P < 0.001. **i** Representative TEM images showing reduced heterochromatin and increased abnormal mitochondria in the *FOS*-knockdown hMSCs. Scale bar, 500 nm. Nu nucleus, Mito mitochondrion. Arrow head denotes the heterochromatin. **j** Analysis of luciferase activity by *in vivo* imaging system (IVIS) showing accelerated decay of *FOS*-knockdown hMSCs after transplantation to TA muscles (left). Luciferase activities were imaged and quantified at days 0, 1, 3, and 5 after transplantation (right). Data were presented as mean ± SD, *n* = 3, **P < 0.01, ***P < 0.001. See also Supplementary Figure S12

To test whether IARGs or CARGs could potentially function downstream of ATF6 in regulating hMSC aging, we first examined the expression of IARGs and CARGs in early and late passage hMSCs. qPCR analysis indicated that none of IARGs were silenced in normally cultured *ATF6*^−/−^ hMSCs relative to WT cells (Supplementary Figure S12A), suggesting that expression of IARGs is only sensitive to ER stress (e.g., TM treatment). Consistent with the definition, CARGs, such as *FOS* and *CDC25B*, were downregulated in cultured *ATF6*^−/−^ hMSCs at both early and late passages (Fig. 5a and 6a). Apart from the basal expression level, we also observed reduced induction of FOS protein in *ATF6*^−/−^ hMSCs upon serum stimulation (Supplementary Figure S12C). The decreased expression of FOS was associated with a lower H3K4me3 level at the promoter of *FOS* in ATF6-deficient hMSCs (Supplementary Figure S12D). To know whether diminishment of CARGs could partially contribute to accelerated senescence in hMSCs, we performed shRNA-mediated knockdown experiments. Among the CARGs (some IARGs included as controls) examined, knockdown of *FOS* (Supplementary Figure S12E) led to the most dramatic effect on repressing hMSC growth (Supplementary Figure S12F). hMSCs expressing *FOS* shRNA displayed typical features of cellular senescence, including decreased percentage of proliferative cells (Fig. 6b–d), increased SA-β-Gal staining positive cells (Fig. 6e), and expression of aging-related molecular signatures (Fig. 6f). Strikingly, the major cellular defects present in *ATF6*^−/−^ hMSCs, including morphological abnormalities in ER, NE, and mitochondria, increased protein aggresomes, and heterochromatin disorganization were also observed in FOS-deficient hMSCs (Fig. 6g–i, Supplementary Figure S12G–I, Supplementary Movie 3, 4). Finally, knockdown of *FOS* resulted in accelerated hMSC attrition in an in vivo niche (Fig. 6j). Collectively, these results indicate that downregulated expression of FOS and perhaps together with other CARGs in hMSCs contributed to accelerated stem cell decay.

## Discussion

By genome editing and directed differentiation, we obtained isogenic *ATF6*^−/−^ hESCs, hMSCs, and hWAPCs. Wild-type and ATF6-deficient hESCs, as well as their differentiation derivatives, constitute a valuable experimental model for studying the biological role of human ATF6. In contrast to hESCs and hWAPCs that do not show noticeable phenotypic changes, ATF6-null hMSCs progressively show functional decay as evidenced by degeneration of membrane organelles, cellular senescence, compromised differentiation ability towards bone and cartilage, etc. A previous murine study failed to identify any aging-associated cellular phenotypes in ATF6-deficident mouse embryonic fibroblasts (MEFs)^36^, suggesting that ATF6’s geroprotective function might be tissue-specific and/or differentiation stage-specific. Our study therefore establishes an important molecular switch linking loss of proteostasis and stem cell aging/exhaustion. Notably, Kohl et al. has recently reported that patients with loss-of-function mutations in *ATF6* from 10 independent families develop an aging-associated disease, cone dysfunction disorder achromatopsia^42^.

ATF6 acts as a sensor/transducer of ER stress. Following ER stress, it translocates from the ER, Golgi, to nucleus and subsequently activates the transcription of downstream genes encoding UPR proteins. Systematic identification of ATF6 target genes undoubtedly represents an important aspect of understanding the essence of cellular homeostasis regulation. So far, the known ATF6 target genes were mainly identified in lower organisms (i.e., *Caenorhabditis elegans*), rodents, or transformed human cell lines^36, 37, 41, 43^. However, these studies failed to identify any constitutive ATF6 target genes in the absence of ER stressor. In addition, no ATF6 target gene has been identified in human diploid cells, including human stem cells and their derivatives. By comparative transcriptomic analysis, we have discovered for the first time hundreds of CARGs and IARGs in three types of human diploid cells (hESCs, hMSCs, and hWAPCs). Of note, ATF6 regulates a large number of CARGs linked to organelle homeostasis maintenance as well as IARGs in hMSCs, relative to hESCs and hWAPCs, which is consistent with the more pronounced phenotypic defects observed in ATF6-null hMSCs. Moreover, very few ATF6 responsive genes are shared among hMSCs, hESCs, and hWAPCs, suggesting that ATF6 exerts different functions in different human diploid cell types. A series of novel ATF6-target genes with classical or non-classical ERSE elements were also revealed in this study, which include mitochondrial factors, cell cycle regulators, calcium modulators, as well as various organelle structural proteins (i.e., integral components of membrane), etc. All these new ATF6 responsive genes constitute useful resources for understanding novel ATF6 biology, extending the current knowledge of ATF6-mediated transcription regulation in human stem cells. In addition, we provide the first evidence showing that ATF6 downregulation mediates hMSC aging through a CARGs-dependent manner, which is independent of classic ER stress-induced ATF6 responsive genes. Given that CARGs are primarily composed of genes encoding for structural components of membrane organelles, these new observations suggest that loss of architectural integrity of membrane organelles may act as a driver of hMSC aging.

Regarding novel cellular phenotypes, our study revealed for the first time that absence of ATF6 in hMSCs resulted in coordinated disorganization of various ER-associated membrane organelles. There are several possible explanations: First, a number of membrane/transmembrane proteins themselves were constitutive ATF6 regulated genes. Downregulation of these proteins as a result of ATF6 deficiency probably leads to the destabilization of membrane organelles. Second, ATF6 is essential for the maintenance of cellular proteostasis, and inactivation of ATF6 in hMSCs may result in the accumulation of protein aggregates , which in turn undermine the integrity of various membrane organelles. Third, ATF6 is essential to maintain the homeostasis of ER, which physically interacts with other membrane organelles, including NE and mitochondria, etc^12–14^. Impairment of ER in ATF6-null hMSCs may lead to structural and/or functional impairment of other adjacent organelles physically connected to ER. How ATF6 coordinates the dynamic interplays between ER, NE, mitochondria, and potentially other membrane organelles during human cellular aging represents an interesting topic that warrants future studies.

**Fig. 7:**
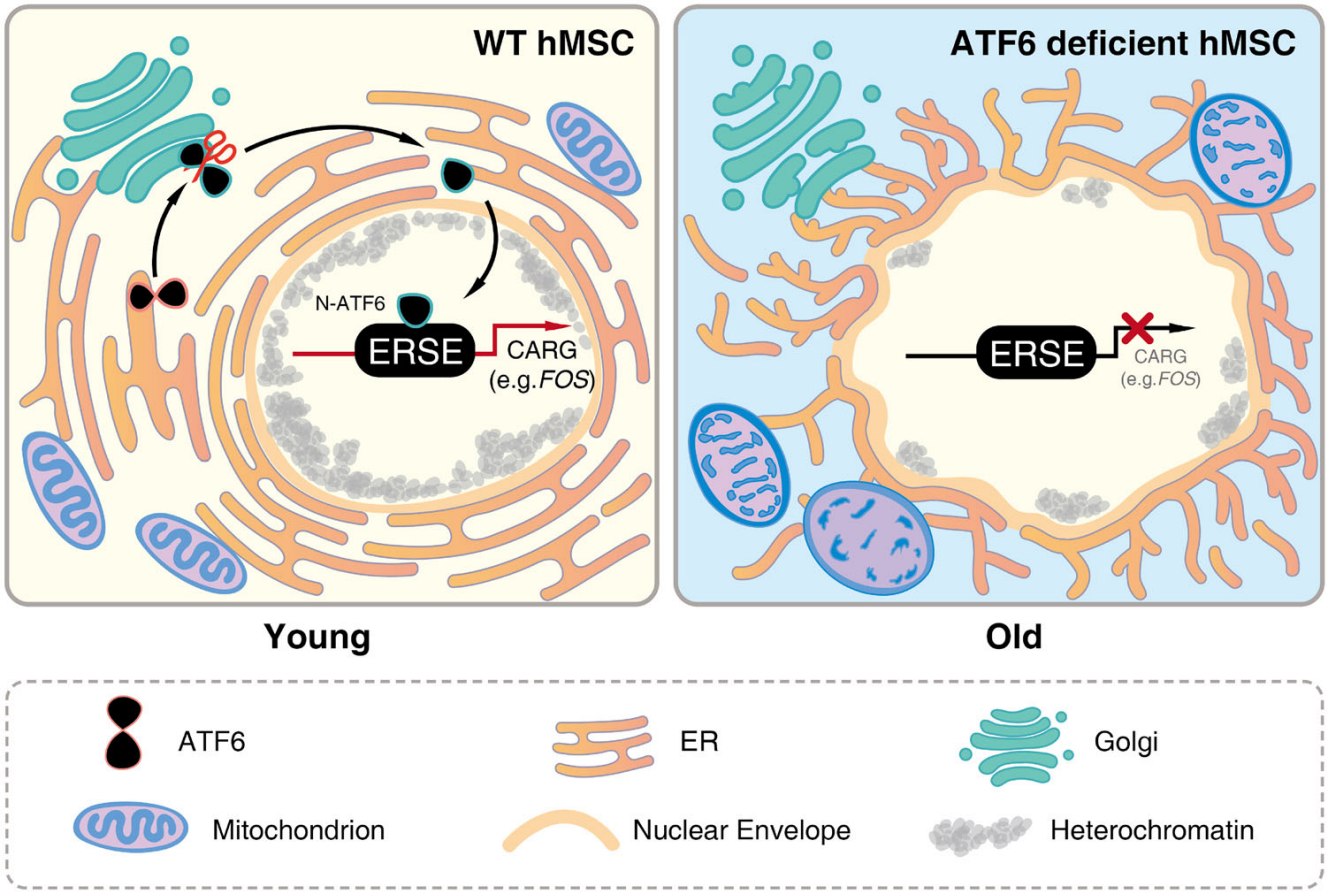
A working model for ATF6 in the homeostasis regulation of ER and ER-associated membrane organelles in hMSCs. In wild-type (WT) hMSCs, transcription factor ATF6 is required to activate an array of constitutive ATF6 responsive genes (CARGs) including *FOS* to maintain the architectural and functional homeostasis of ER, mitochondria, nuclear envelope, and heterochromatin, and safeguard cells from premature aging. Deficiency of ATF6 diminishes the expression of CARGs, results in coordinated impairment of membrane organelles, and leads to accelerated senescence of hMSCs.

Concerning molecular mechanisms, *FOS* was identified as a novel ATF6 target gene in hMSCs. *FOS* encodes leucine zipper protein that can dimerize with proteins of the JUN family, thereby forming the transcription factor complex AP-1^44, 45^. ATF6 binds to a typical ERSE present at *FOS* promoter to activate transcription. Downregulation of *FOS* in hMSCs recapitulated major cellular defects including organelle degeneration and cellular senescence observed in ATF6-deficient hMSCs. While the details of how *FOS* regulates hMSC homeostasis have not been revealed, several reports suggest a link between FOS and aging. First, expression of *FOS* was compromised in senescent human fibroblasts^46, 47^. Second, mutations in *Fos/Jun* genes were demonstrated during epidermal aging^48^. Third, Fos knockout mice exhibited a shortened life span of 6–7 months with growth retardation and severe osteoporosis^49^. These observations, together with our findings, indicate an involvement of FOS in regulating hMSC aging, as a downstream effector of ATF6 (Fig. 7).

In sum, we expand our knowledge of ATF6-regulated gene network in human diploid cells, and uncover a novel role of ATF6 in regulating human organelle homeostasis and stem cell aging. The new factors and pathways identified may be targeted to develop new treatment for aging and age-related disorders.

## Experimental procedures

### Cell culture

H9 hESCs (WiCell Research) and ATF6-deficient hESCs were maintained on Mitomycin C-inactivated MEF or Matrigel-coated plates as previously described^27, 29, 32–34^. hMSCs were cultured in αMEM (Invitrogen) medium supplemented with 10% fetal bovine serum (FBS, Gemcell), 1% penicillin/streptomycin (Gibco), 0.1 mM non-essential amino acids (Gibco), and 1 ng/ml bFGF (Joint Protein Central, JPC). HEK293T cells were cultured in high glucose DMEM (Hyclone) containing 10% FBS.

### Animal experiments

All animal experiments performed in this study were approved by the Chinese Academy of Science Institutional Animal Care and Use Committee. For teratoma formation assay, 6–8-week-old male NOD-SCID mice were injected s.c. with 3 × 10^6^ WT or ATF6-deficient hESCs in a Matrigel/mTeSR solution, respectively. Teratomas were collected when reaching a size of around 10 mm in diameter, and subjected to immunostaining^50, 51^. For hMSC transplantation assay, 10^6^ hMSCs (WT and ATF6 deficient, or with FOS knockdown) expressing luciferase were injected to TA muscle of 6–8-week-old male mice. Mice were imaged in vivo with IVIS spectrum imaging system (XENOGEN, Caliper) for luciferase activity. For western blotting assay, female C57 mice were anesthetized and heart perfusion was performed with cold PBS. After the perfusion, thoracic aortas from three young (6-week-old) and three old (15-month-old) mice were carefully dissected and immediately placed in liquid nitrogen for later experiments. Each cage contained animals of all the different experimental groups. Exclusion criteria such as inadequate injection or died during experiments due to technical problems were pre-determined.

### Generation of ATF6^−/−^ hESCs

CRISPR/Cas9-mediated gene targeting was performed as previously described^52^. In brief, a donor plasmid was constructed by including 1.0–2.0-kb homology arms and drug resistance cassettes (neo). H9 hESCs (5.0 × 10^6^) were electroporated with gRNA vectors and the Cas9 plasmid, and subsequently cultured on DR4 MEF feeder cells. G418 (100 μg/ml, Invitrogen) was then added to the medium to initiate positive selection 2–4 days after electroporation. After about 2 weeks’ drug selection, G418-resistant clones were manually picked and transferred to a 96-well plate for further expansion. Correctly gene-targeted clones were identified by qRT-PCR and western blotting. Primers were listed in Supplementary Table S1.

### hMSC generation and characterization

Differentiation of WT and ATF6-deficient hMSCs from hESCs was carried out as previously described^27, 29, 32, 34^. Briefly, embryoid bodies were plated on Matrigel in differentiation medium (αMEM (Invitrogen) medium supplemented with 10% FBS (Gemcell), 5 ng/ml TGFβ (Human Zyme), 10 ng/ml bFGF (JPC), and 1% penicillin/streptomycin (Gibco)). About 10 days later, differentiated cells were passaged once. To further purify hMSCs, the differentiated cells were then subjected to fluorescence activated cell sorting (FACS) by evaluating the hMSC-specific surface markers (CD73, CD90, and CD105). The antibodies of hMSC-specific markers used in FACS were as follows: anti-CD90-FITC (BD Bioscience, 555595), anti-CD73-PE (BD Bioscience, 550257), and anti-CD105-APC (BD Bioscience, 17-1057-42). Anti-IgG-FITC (BD Biosciences, 555748), anti-IgG-PE (BD Biosciences, 555749), and anti-IgG-APC (BD Biosciences, 555751) antibodies were also used as isotype controls. The differentiation potentials of hMSC towards osteoblasts, chondrocytes and adipocytes were verified by histochemical staining with von Kossa (osteogenesis), Alcian blue (chondrogenesis), and Oil red O (adiopogenesis) Kit (IHC World), respectively.

### SA***-***β***-***Gal staining assay

Cells were stained according to a previously described method^27, 29, 34^.

### Clonal expansion assay

About 2000 cells were seeded in each well of 12-well plates, cultured for 2 weeks, and stained with 0.2% crystal violet. Colony numbers were counted using light microscope in randomly selected fields. Each experiment was performed in three independent replicates.

### Bisulfite sequencing of the *OCT4* promoter

Bisulfite conversion of DNA was carried out using CpGenome Fast DNA Modification Kit (Millipore) according to manufacturer’s protocol. A genomic fragment of the *OCT4* promoter was amplified using LA Taq Hot StartVersion (TAKARA). In brief, PCR products were purified by gel extraction using QIAquick columns (Qiagen), and then cloned into the pEasy-T1 vector (Transgen). Eight clones from each sample were sequenced with the universal primer M13.

### Transmission electron microscopy

Cells were fixed with a solution containing 2.5% glutaraldehyde overnight at 4 °C. After fixation, the samples were washed with 0.1 M sodium phosphate buffer for three times, and were post-fixed in 1.0% osmium tetroxide for 1 h. Then the samples were blocked with 1% uranyl acetate, dyed for 1 h, and dehydrated through a graded series of acetone to 100% and placed into liquid Spurr epoxy resin for saturating overnight at 37 °C. Then, the samples were polymerized in a 65 °C oven for 2 days and the embedded samples were cut using Leica ultramicrotome EM UC6 (Leica). Later, the samples were stained with uranyl acetate and lead citrate in a Leica EM Stainer and imaged under a Spirit Transmission Electron Microscope (FEI Company) operating at 120 kV.

### Structured illumination microscopy

*Imaging condition*: All images were acquired by total internal reflection fluorescence structured illumination microscopy (TIRF-SIM) system^53^ with the Olympus 1.49-NA objective under the physiological conditions of 37 °C and 5% CO_2_. At each time point, we acquired three raw images at successive phase steps of 0, 1/3, and 2/3 of the illumination period. We then repeated this process with the standing wave excitation pattern rotated ± 120° with respect to the first orientation, for a total of nine raw images. The film speed of rebuild image is 1 frame per second. The excitation intensities is 40 W/cm^2^.

*TIRF-SIM system*: The TIRF-SIM system used was as described previously^53^. TIRF-SIM was built on an inverted fluorescence microscope (Olympus IX83, Japan). The beam from a laser combiner equipped with 488 nm (500 mW, Coherent, Genesis Max 488-500 STM) and 560 nm (1 W, MPB Communications, VFL-P-500-560) lasers is passed through an acousto-optic tunable filter (AOTF; AA Quanta Tech, AOTFnc-400.650-CPch-TN). The beam is then expanded to a 1/e^2^ diameter of 12 mm and sent to a phase-only modulator^54^ consisting of a polarizing beam splitter, a achromatic half-wave plate (HWP; Bolder Vision Optik, BVO AHWP3), and a ferroelectric spatial light modulator (SLM; Forth Dimension Displays, SXGA-3DM). In the imaging of hMSCs, the light diffracted by the grating pattern displayed on SLM passes through a polarization rotator^55^ consisting of a liquid crystal cell (LC; Meadowlark, SWIFT) and an achromatic quarterwave plate (QWP; Bolder Vision Optik, BVOAQWP3), which rotates the linear polarization of the diffracted light so as to maintain the s-polarization necessary to maximize the pattern contrast for all pattern orientations. A mask consisting of a hollow barrel with slots for different pattern orientations^55^ is driven by a galvanometric scanner (Cambridge Technology, 6230HB) to filter out all diffraction orders created by the binary and pixelated nature of the SLM except for the desired ± 1 diffraction orders. These are then imaged at the back focal plane of the objective (Olympus, UAPON100XOTIRF 1.49 NA) as two spots at the opposite sides of the pupil. After passage through the objective, the two beams intersect at the interface between the coverslip and the sample at an angle exceeding the critical angle for total internal reflection. An evanescent standing wave penetrating ~100 nm into the sample is thereby generated, consisting of a sinusoidal pattern of excitation intensity that is a low-pass filtered image of the SLM pattern. The period, orientation, and relative phase of this excitation pattern can be finely tuned by altering the corresponding pattern displayed on SLM. For each orientation and phase of the applied excitation pattern, the resulting fluorescence is collected by the objective, focused by a tube lens at an intermediate image plane, separated from the excitation light by a dichroic mirror (Chroma, ZT488/561tpc_22.5 deg) placed between two relay lenses, and reimaged onto a sCMOS camera (Hamamatsu, Orca Flash 4.0 v3 sCMOS), where the structured fluorescence emission pattern is recorded.

*Cell culture*,* transfection*,* and imaging*: hMSCs were transiently transfected with KDEL-mCherry vector using Lipofectamine 3000 (Thermo Fisher Scientific) according to the manufacturer’s instructions. After 24 h, the transfected cells were plated on 25-mm coverslips, pre-coated with 10 mg/ml fibronection (Millipore, FC010). Twelve hours after plating, hMSCs were changed with fresh medium and then the imaging was performed in a humidified atmosphere with 5% CO_2_ at 37 °C. A cell is considered as an abnormal one when over 60% of its ER’s area displays unbranched morphology.

### Calcium imaging

Calcium imaging assay was performed as previously described^56^. Briefly, cells grown on the coverslips were loaded with ratiometric Ca^2+^ indicator dye Fura-2 AM (Thermo, F1221), which was facilitated by Pluronic F-127 (Thermo, P3000MP) for solubilization in KRH calcium imaging buffer (dilute 25 ml 5 M NaCl, 25 ml 1 M HEPES, 12 ml 0.5 M Glucose, 2 ml 2.5 M KCl, 3 mL 0.2 M KH_2_PO4, 1.2 ml 1 M MgCl_2_, 1 ml 2 M Ca^2+^ with ddH_2_O to 1 L, and adjust pH to 7.3 by NaOH). After being loaded for 30 min at room temperature (RT) (away from light), the coverslip was washed with KRH buffer for three times and then subjected to imaging on a perfusion chamber on an inverted Nikon TiE microscope with 20× Fluar objective. The Metafluor Program software was used to monitor the calcium concentration changes in the cytoplasm, and the intracellular Ca^2+^ concentration was expressed as the 340/380 ratio. When beginning the test, the imaging buffer was perfused and changed from 2 mM Ca^2+^ to 0 mM Ca^2+^ (with 1 mM EGTA) buffer followed by 0 mM Ca^2+^ KRH buffer (1 mM EGTA) with CPA (20 μM, Sigma, C1530) to deplete the store of ER, and then changed to 2 mM Ca^2+^ buffer with CPA (20 μM) to induce store-operated calcium entry (SOCE) response. The Fura-2 amplitude changes of store and SOCE for each cell line were calculated and compared.

### qRT-PCR

Total RNA was extracted using TRIzol Reagent (Invitrogen) and 1–2 μg total RNA was used for cDNA synthesis with reverse transcription Master Mix (Promega). Quantitative real-time PCR was performed with iTaq Universal SYBR Green Super mix (Bio-Rad) on a CFX384 Real-Time PCR system (Bio-Rad). All data was normalized with 18S rRNA transcript and calculated using the ΔΔCq method. All qRT-PCR primer pairs are listed in Supplementary Table S1.

### Western blotting

Protein quantification was performed using a BCA Kit. Protein lysates was subjected to SDS-PAGE and subsequently electro-transferred to a polyvinylidene fluoride membrane (Millipore). Western blotting was performed as previously described^57, 58^. The primary antibodies used were as follows (company, catalog number): anti-ATF6 (Santa Cruz, sc-22799), anti-BIP (Cell Signaling Technology, 3177), anti-P16 (BD Bioscience, 550834), anti-P21 (Cell Signaling Technology, 2947), anti-Lamin B, anti-LAP2 (BD Bioscience, 611000), anti-FOS (Cell Signaling Technology, 2250), anti-Calreticulin (Abcam, ab2907), anti-Calnexin (Cell Signaling Technology, 2679), anti-Ero1Lα (Cell Signaling Technology, 3264), anti-IRE1 (Cell Signaling Technology, 3294), anti-PDI (Cell Signaling Technology, 3501), anti-PERK (Cell Signaling Technology, 5683), anti-EMDM (Santa Cruz, sc-377394), anti-β-Actin (Santa Cruz, sc69879), anti-GAPDH (Santa Cruz, sc-25778), and anti-Flag (Sigma, F1804).

### Immunofluorescence

Cells were fixed with 4% paraformaldehyde for 25 min, permeabilized with Triton X-100 (0.3% in PBS) for 25 min, incubated with blocking buffer (10% donkey serum in PBS) for 1 h at RT, and stained with primary antibodies overnight at 4 °C. Then, the cells were incubated with secondary antibodies for 1 h at RT. Hoechst 33342 (Invitrogen) was used to stain nuclear DNA. The primary antibodies used in immunofluorescence assays were as follows: anti-NANOG (Abcam, ab21624), anti-SOX2 (Santa Cruz, sc-17320), anti-OCT4 (Santa Cruz, sc-5279,), anti-SMA (Sigma, A5228), anti-TUJ1 (Sigma, T2200), anti-FOXA2 (Cell Signaling Technology, 8186S), anti-BIP (Cell Signaling Technology, 3177), anti-LaminA/C (Santa Cruz, sc-6215), anti-Calreticulin (Abcam, ab2907), anti-LaminB (Santa Cruz, sc-6217), anti-LAP2 (BD Bioscience, 611000), and anti-Ki67 (ZSGB-BIO, ZM0166).

### Flow cytometry analysis

For cell cycle analysis, hMSCs were collected and fixed in 70% ice-cold ethanol at 4 °C overnight. Next day, the cells were stained with 0.02 mg/ml propidium iodide and 0.2 mg/ml RNase at 37 °C for 30 min after several washes with PBS. Then the cells were later analyzed using BD LSRFortesa, and cell-cycle phase distributions were analyzed by ModFit software. For aggresome intensity analysis, the cells were collected and then stained using PROTEOSTAT® Aggresome detection kit according to the manufacturer’s instructions (ENZ-51035-K100, ENZO). For MMP analysis, the cells were collected and then stained with Cell Meter™ JC-10 Mitochondrial Membrane Potential Assay Kit (22801, AAT Bioquest®, Inc.).

### Plasmids

The cDNAs of Flag-ATF6-CA and Flag-ATF6-ΔTAD were cloned into pLE4 lentiviral vector (a gift from Dr. Tomoaki Hishida). Primers used are listed in Supplementary Table S1. Flag-luciferase expression vector was obtained as previously described^34^. pGL3-Basic and pGL3-Promoter vectors were purchased from Promega. The promoters of ATF6 regulated genes were cloned into pGL3-Basic vector. The putative ERSE-contained sequence of *FOS* was also cloned into pGL3-Promoter vector. Cloning primers are also listed in Supplementary Table S1. pCDNA 3.1 and pLVTHM vectors were purchased from Invitrogen and Addgene, respectively.

### Luciferase reporter assay

For luciferase reporter assay, hMSCs were culture in 24-well plates and co-transfected with 0.5 μg plasmid containing luciferase driven by different promoters, 0.2 μg renilla, and 0.5 μg pcDNA3.1 or 0.5 μg pcDNA3.1-ATF6-CA or pcDNA3.1-ATF6-ΔTAD expression vector using Lipofectamine® 3000 (Invitrogen) following the manufacturer’s protocol. Forty-eight hours after transfection, cells were collected and relative luciferase activity was measured using Dual-Luciferase Reporter Assay System (Promega).

### RNA interference of CARGs and IARGs by shRNAs

The shRNAs were introduced into hMSCs by lentivirus which was produced from the HEK293T cells by co-transfection with the transfer vector pLVTHM and packaging plasmids psPAX and pMD2.G. The sequence information of shRNAs targeting the coding sequence of several CARGs and IARGs genes are shown in the Supplementary Figure S12F. The sense and antisense sequences were annealed, and inserted into the lentiviral transfer vector pLVTHM.

### Lentivirus production

Lentivirus particles were generated from HEK293T cells as previously described^27, 34^. Briefly, HEK293T cells were co-transfected with transfer plasmid and packaging plasmids by Lipofectamine 3000 transfection reagent (Invitrogen). Viral particle-containing supernatants were harvested 48 and 72 h later and were concentrated by ultra-centrifugation (20,000×*g* for 2.5 h), and later used for infecting hMSCs in the presence of 10 μg/ml polybrene.

### RNA-seq library construction

One million cells were used to extract total RNA using the RNeasy Mini Kit (Qiagen) according to the manufacturer’s protocol. After quantification of RNA by Fragment Analyzer (Advanced Analytical), 1.5 μg of total RNA was used to construct sequencing libraries by TruSeq RNA Sample Preparation Kit (Illumina) according to the manufacturer’s instructions.

### ChIP-seq and ChIP-qPCR

ChIP-seq and ChIP-qPCR were performed according to a published protocol^59^. Briefly, cells were crosslinked and lysed, and then the chromatin was sheared into fragments using sonication. To obtain fragments of interest, samples were incubated with anti-H3K4me3 antibody (Abcam, ab8580) or anti-Flag (Sigma, F1804) antibody overnight. Inputs served as negative controls. After the DNA was de-crosslinked and extracted, ChIP-seq and qPCR were conducted as previously described^27, 29, 33, 34^. The sequencing library was constructed by TruSeq DNA Sample Preparation Kit (Illumina) or NEBNext® DNA Library Prep Reagent Set (NEB) following the manufacturer’s protocol. The primers’ sequences for ChIP-qPCR are listed in Supplementary Table S1.

### Sequencing data quality control

All of the de-multiplexed sequencing reads were first cleaned to remove any artificial sequences, such as sequencing adapters introduced during the experimental process, and reads with more than 10% low-quality bases were also discarded.

### RNA-seq data processing

For the RNA-seq data, the annotation of the transcriptome was defined as UCSC hg19 RefSeq genes. Then the cleaned reads were aligned to the hg19 reference genome using TopHat (version 2.0.9)^60^ with the default parameters. HTSeq^61^ were used to count the number of reads mapped in each annotated gene based on the mapping results. This results were further used for the calculation of differentially expressed genes using DESeq2^62^ package in bio-conductor. Furthermore, for the quantification of RNA-expression, the gene expression levels (FPKM) of each sample were calculated using cufflinks (v2.2.1).

### ChIP-seq data processing

The whole ChIP-seq analysis pipeline was available at https://github.com/huboqiang/ChIP. In brief, for the ChIP-seq data, the trimmed reads were mapped to the human genome (hg19 assembly) using BWA aligner (version 0.7.5a-r405) with the options “−i 15 −q 10 −t 4”^63^. Only the unique mapped reads were retained for further analyses. The density track and peaks were called by MACS2 (v2.10)^64^ based on the mapping results. The density track of interval *i* in each sample was further normalized by

$${\rm normalized}_{{\rm density}_i} = \frac{{{\rm treat}\_{\rm density}_i}}{{\mathop {\sum }\nolimits_k {\rm treat}\_{\rm density}_i \times 10^6}} \\ - \frac{{{\rm control}\_{\rm density}_i}}{{\mathop {\sum }\nolimits_k {\rm control}\_{\rm density}_i \times 10^6}}.$$

In order to get rid of the technical noises, two replications were performed for ChIP-Seq data. The peaks were called using the Irreproducibility Discovery Rate (IDR) framework developed by Kundaje (https://sites.google.com/site/anshulkundaje/projects/idr)^65^ and the densities were calculated using the merged results for samples which passed the IDR framework.

### Statistical analysis

The results were presented as mean ± SEM or mean ± SD. Two-tailed Student’s *t*-test was used to compare differences between treatments assuming equal variance, and conducted using Graph-Pad Prism Software. *P* values <0.05, <0.01, and <0.001 were considered statistically significant (*, **, ***).

### Accession numbers

All of the RNA-seq and ChIP-seq data have been deposited in GEO under the accession number GSE102004.

## Electronic supplementary material

Supplementary Information

Supplementary Movie 1

Supplementary Movie 2

Supplementary Movie 3

Supplementary Movie 4

Supplementary Table 1

Supplementary Table 2

Supplementary Table 3

Supplementary Table 4

Supplementary Table 5

Supplementary Table 6

Supplementary Table 7

Supplementary Table 8

Supplementary Table 9
