# Supplementary material for: ATF6 safeguards organelle homeostasis and cellular aging in human mesenchymal stem cells
Source: Cell Discov. 2018 Jan 5;4:2. doi: 10.1038/s41421-017-0003-0 (PMC5798892; doi:10.1038/s41421-017-0003-0)
Supplement: Supplementary file 1 — Supplementary Information [file 41421_2017_3_MOESM1_ESM.pdf]

Supplementary Information

Supplementary Figures and Figure Legends

Figure S1

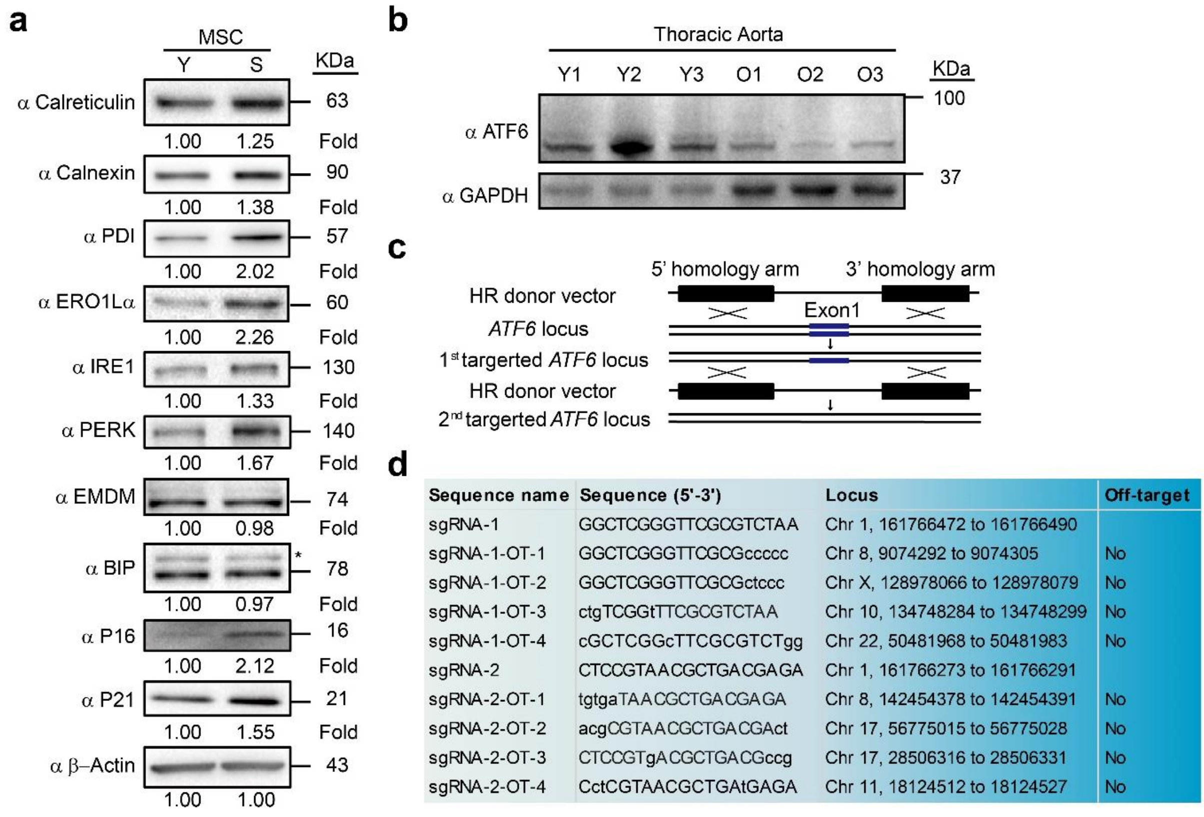

Sequence name	Sequence (5'-3')	Locus	Off-target
sgRNA-1	GGCTCGGGTTCGCGTCTAA	Chr 1, 161766472 to 161766490	
sgRNA-1-OT-1	GGCTCGGGTTCGCGccccc	Chr 8, 9074292 to 9074305	No
sgRNA-1-OT-2	GGCTCGGGTTCGCGctccc	Chr X, 128978066 to 128978079	No
sgRNA-1-OT-3	ctgTCGGtTTCGCGTCTAA	Chr 10, 134748284 to 134748299	No
sgRNA-1-OT-4	cGCTCGGcTTCGCGTCTgg	Chr 22, 50481968 to 50481983	No
sgRNA-2	CTCCGTAACGCTGACGAGA	Chr 1, 161766273 to 161766291	
sgRNA-2-OT-1	tgtgaTAACGCTGACGAGA	Chr 8, 142454378 to 142454391	No
sgRNA-2-OT-2	acgCGTAACGCTGACGAct	Chr 17, 56775015 to 56775028	No
sgRNA-2-OT-3	CTCCGTgACGCTGACGccg	Chr 17, 28506316 to 28506331	No
sgRNA-2-OT-4	CctCGTAACGCTGAtGAGA	Chr 11, 18124512 to 18124527	No

Supplementary Figure S1. Generation and characterization of ATF6-deficient hESCs. a Western blotting analysis of the expression of UPR-relative proteins in young and replicative senescent hMSCs. β-Actin was used as the loading control. Asterisk stands for the non-specific band. Y, young, S, senescent. **b** Western blotting showing the decreased ATF6 expression in the thoracic aortas of aged mice. Thoracic aortas from three young (6-week-old) and three old (15-month-old) mice were collected and then subjected to western blotting. GAPDH was used as the loading control. **c** Schematic representation of knockout of *ATF6* by exon 1 removal via CRISPR/Cas9-faciliated homologous recombination (HR). **d** Predicted off-target sites (above 14 nucleotides matched) against sgRNA1 and sgRNA2 were shown in the table. No off-target cleavage was found both in the *ATF6*$^{-/-}$ #1 and *ATF6*$^{-/-}$ #2 hESCs. Mismatched nucleotides compared to *ATF6* locus were highlighted with lower case. Primers used to amplify the predicted off-target sites were listed in Supplementary Table S1. OT, off-target.

Figure S2

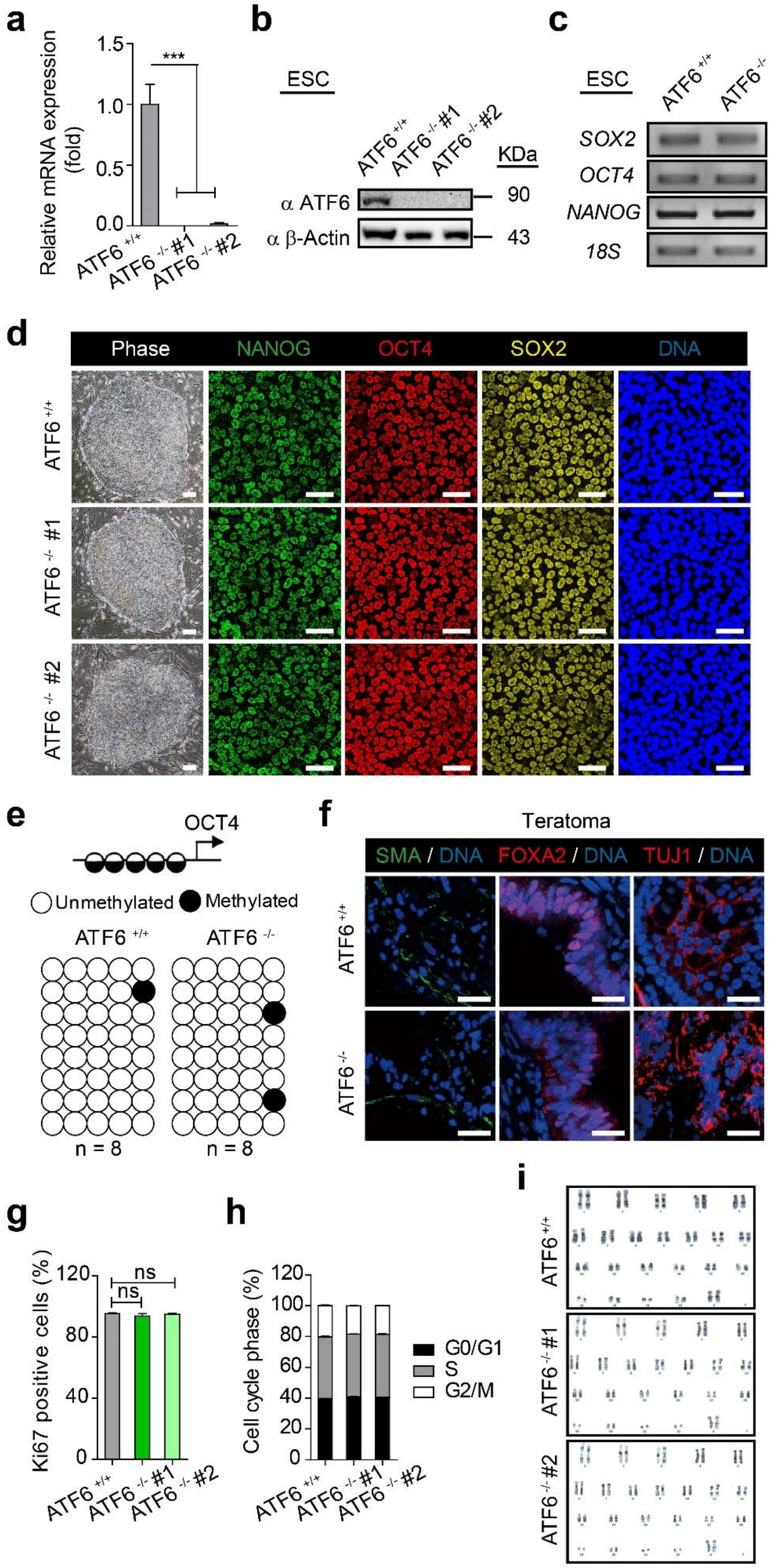

a
Relative mRNA expression (fold)
1.5
1.0
0.5
0.0
***
ATF6 +/+
ATF6 -/- #1
ATF6 -/- #2
b
ESC
ATF6 +/+
ATF6 -/- #1
ATF6 -/- #2
KDa
α ATF6
90
α β-Actin
43
c
ESC
ATF6 +/+
ATF6 -/-
SOX2
OCT4
NANOG
18S
d
Phase
NANOG
OCT4
SOX2
DNA
ATF6 +/+
ATF6 -/- #1
ATF6 -/- #2
e
OCT4
Unmethylated
Methylated
ATF6 +/+
ATF6 -/-
n = 8
n = 8
f
Teratoma
SMA / DNA
FOXA2 / DNA
TUJ1 / DNA
ATF6 +/+
ATF6 -/-
g
Ki67 positive cells (%)
120
80
40
0
ns
ns
ATF6 +/+
ATF6 -/- #1
ATF6 -/- #2
h
Cell cycle phase (%)
120
100
80
60
40
20
0
G0/G1
S
G2/M
ATF6 +/+
ATF6 -/- #1
ATF6 -/- #2
i
ATF6 +/+
ATF6 -/- #1
ATF6 -/- #2

Supplementary Figure S2. Generation and characterization of ATF6-deficient hESCs. a qPCR analysis of ATF6 mRNA in WT and ATF6-deficient hESCs. A pair of qPCR primers targeting the region of *ATF6* mRNA exon 1 was used. Data were presented as mean ± SEM, n = 3, ***P < 0.001. **b** Western blotting analysis of ATF6 protein in WT and ATF6-deficient hESCs. β-Actin was used as the loading control. **c-d** RT-PCR and immunofluorescence analysis showing comparable mRNA (c) and protein (d) expression levels of pluripotency markers, including NANOG, OCT4, SOX2 between WT and ATF6-deficient hESCs. Scale bar, 50 μm. **e** The methylation pattern of the promoter of *OCT4* in WT and ATF6-deficient hESCs. **f** Immunostaining analysis of teratomas derived from WT and ATF6-deficient hESCs showing their comparable *in vivo* differentiation potentials to ectodermal (TUJ1 positive cells), mesodermal (SMA positive cells) and endodermal (FOXA2 positive cells) tissues. Scale bar, 100 μm. **g** The percentages of Ki67 positive cells in WT and ATF6-deficient hESCs were compared. Data were presented as mean ± SEM, n = 3, ns, not significant. **h** Cell cycle analysis showing comparable cell cycle phases in WT and ATF6-deficient hESCs. Data were presented as mean ± SEM, n = 3. **i** Karyotyping analysis of WT and ATF6-deficient hMSCs indicating normal karyotypes.

Figure S3

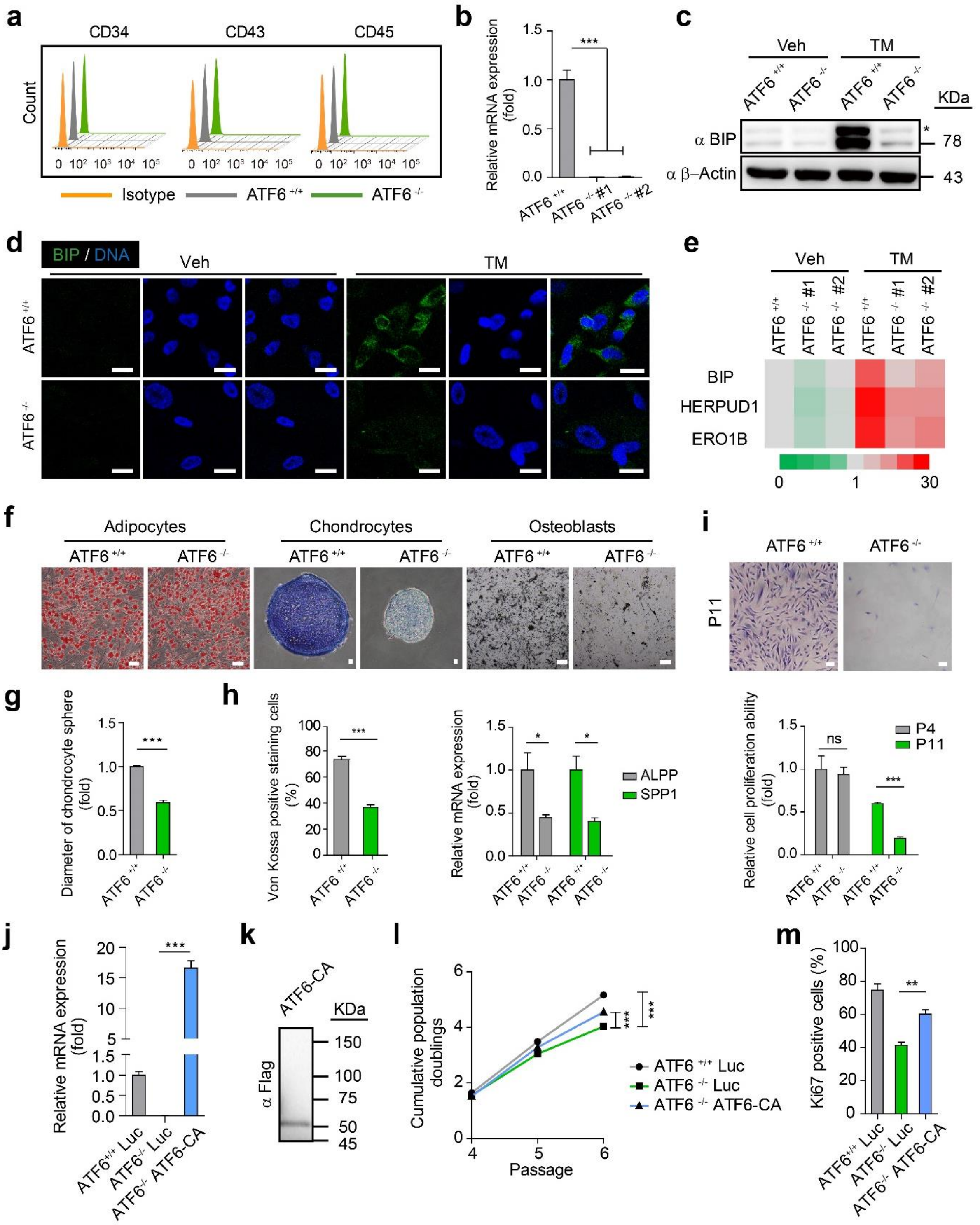

a
CD34
CD43
CD45
Count
Isotype
ATF6 +/+
ATF6 -/-
b
Relative mRNA expression (fold)
***
ATF6 +/+
ATF6 -/- #1
ATF6 -/- #2
c
Veh
TM
KDa
α BIP
α β-Actin
78
43
d
BIP / DNA
Veh
TM
ATF6 +/+
ATF6 -/-
e
Veh
TM
BIP
HERPUD1
ERO1B
0
1
30
f
Adipocytes
Chondrocytes
Osteoblasts
i
P11
g
Diameter of chondrocyte sphere (fold)
h
Von Kossa positive staining cells (%)
Relative mRNA expression (fold)
ALPP
SPP1
Relative cell proliferation ability (fold)
ns
P4
P11
j
Relative mRNA expression (fold)
ATF6+/+ Luc
ATF6-/- Luc
ATF6-/- ATF6-CA
k
ATF6-CA
KDa
α Flag
l
Cumulative population doublings
Passage
ATF6 +/+ Luc
ATF6 -/- Luc
ATF6 -/- ATF6-CA
m
Ki67 positive cells (%)
**

Supplementary Figure S3. Generation and characterization of ATF6-deficient hMSCs. a FACS analysis showing the absence of non-MSC markers CD34, CD43, and CD45 in WT and ATF6-deficient hMSCs. **b** qRT-PCR analysis confirming the absence of *ATF6* mRNA in ATF6-deficient hMSCs. Data were presented as mean ± SEM, n = 3, ***p<0.001. **c-d** Western blotting (c) and immunofluorescence (d) analysis showing the decreased expression of BIP in the ATF6-deficient hMSCs when treatment with TM. β-Actin was used as the loading control. Asterisk denotes the non-specific band. **e** The heatmap showing qRT-PCR analysis of the mRNA expression of *BIP*, *HERPUD1* and *ERO1B* in the WT and ATF6-deficient hMSCs when treated with ER stress inducer tunicamycin (TM). **f** Adiopogenesis, chondrogenesis and osteogenesis potentials of WT and ATF6-deficient hMSCs. Oil Red-O, Alcian blue, and Von Kossa were used to characterize adipocytes, chondrocytes, and osteoblasts, respectively. Scale bar, 20 μm. **g** The diameters of chondrocyte spheres were measured and compared. Data were presented as mean ± SD, n = 9, ***p<0.001. **h** Von Kossa positive cells were calculated and compared in the left panel. Data were presented as mean ± SEM, n = 3, ***p<0.001. qRT-PCR analyses showed reduced expression of osteoblast-specific markers ALPP, SPP1 in the osteoblast derivatives differentiated from ATF6-deficient hMSCs in the right panel. Data were presented as mean ± SEM, n = 3, *p<0.05. **i** Clonal expansion assay showing cell proliferation ability of WT and ATF6-deficient hMSCs at early (EP, Passage 4) and late passages (LP, Passage 11), respectively. Cells were stained with crystal violet after a two-week culture, and the numbers of the crystal violet-positive cells were quantified. Data were presented as mean ± SEM, n = 3, ns, not significant, ***p<0.001. Scale bar, 20 μm. **j** WT and *ATF6*$^{-/-}$ hMSCs were transduced with lentiviral vector encoding a Flag-tagged constitutively active ATF6 (ATF6-CA) or Flag-tagged luciferase (Luc) at passage 4. The mRNA levels of *ATF6* were examined by qRT-PCR at passage 5. **k** Western blotting with anti-Flag antibody showing proper expression of ATF6-CA in the *ATF6*$^{-/-}$ hMSCs transduced with Flag-tagged ATF6-CA at passage 4. The cell lysates were collected at passage 5 for western blotting. **l** Growth curve showing the cumulative population doublings of WT and ATF6-deficient hMSCs transduced with lentiviral vector encoding ATF6-CA or luciferase from passage 4 to passage 6. **m** Increased Ki67 positive cells were observed after overexpression of ATF6-CA in comparison with overexpression of luciferase in *ATF6*$^{-/-}$ hMSCs. Data were shown as mean ± SEM, n = 3, ***p<0.001.

Figure S4

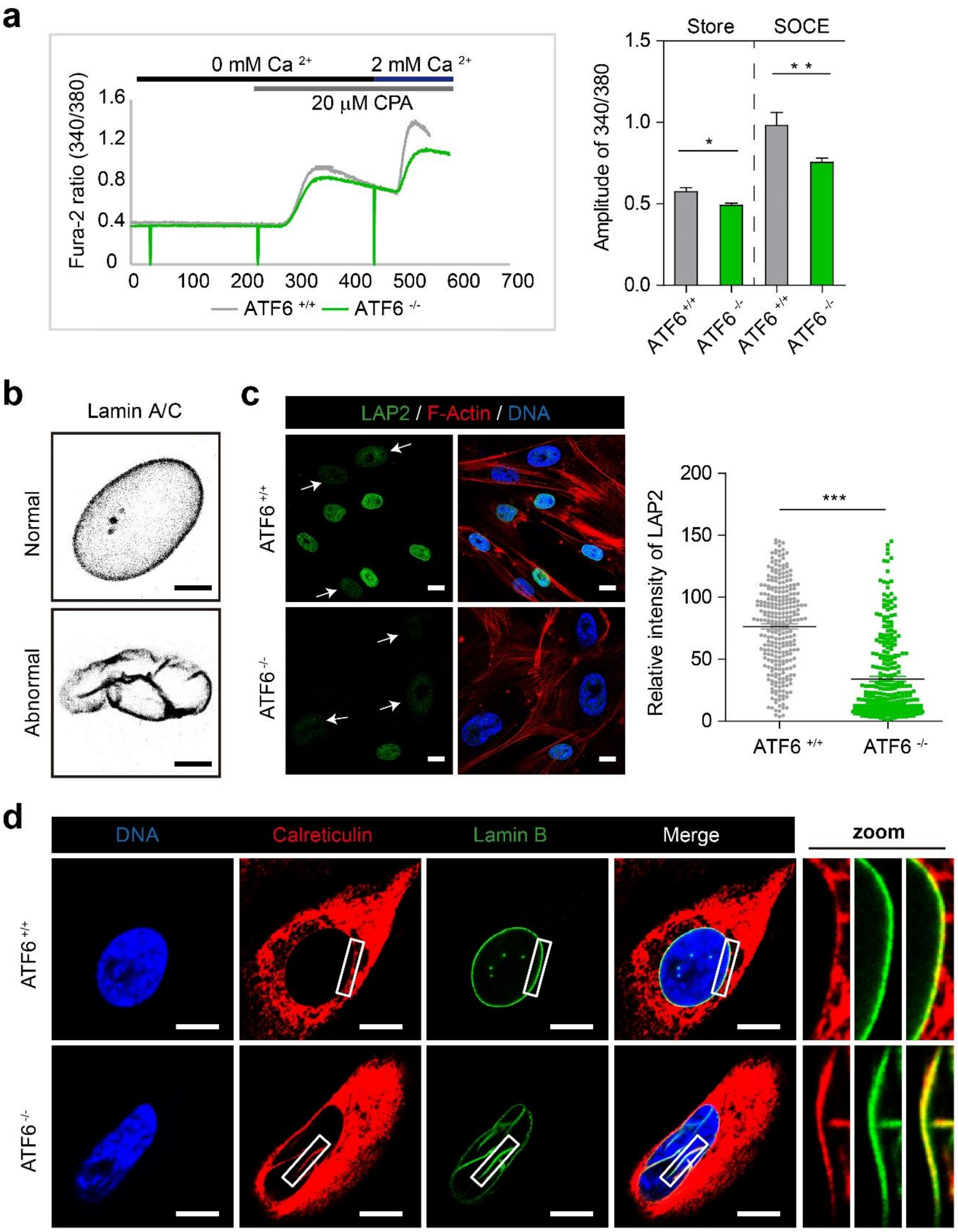

a
0 mM Ca 2+
2 mM Ca 2+
20 μM CPA
Fura-2 ratio (340/380)
ATF6 +/+
ATF6 -/-
Store
SOCE
Amplitude of 340/380
b
Lamin A/C
Normal
Abnormal
c
LAP2 / F-Actin / DNA
Relative intensity of LAP2
d
DNA
Calreticulin
Lamin B
Merge
zoom

Supplementary Figure S4. Disruption of ER and ER-associated membrane organelle homeostasis in ATF6-deficient hMSCs. a Calcium imaging analysis showing the decreased store calcium influx and decreased store-operated calcium entry (SOCE) in the ER of ATF6-deficient hMSCs compared to WT hMSCs. **b** Representative immunostaining images showing the normal and abnormal morphology of nuclear envelop (NE) labelled by Lamin A/C antibody. Scale bar, 5 μm. **c** Representative immunostaining images showing the LAP2 expression in WT and ATF6-deficient hMSCs. Scale bar, 20 μm. The relative intensity of LAP2 in 288 cells was measured with Image J software and the calculated data were shown as mean ± SD, ***P < 0.001. **d** Representative immunostaining images showing the nuclear envelop and ER labelled with Lamin B and Calreticulin, respectively. Scale bar, 10 μm. Zoom pictures on the right are the merged images of white box area in higher magnification showing the overlapping of NE and ER.

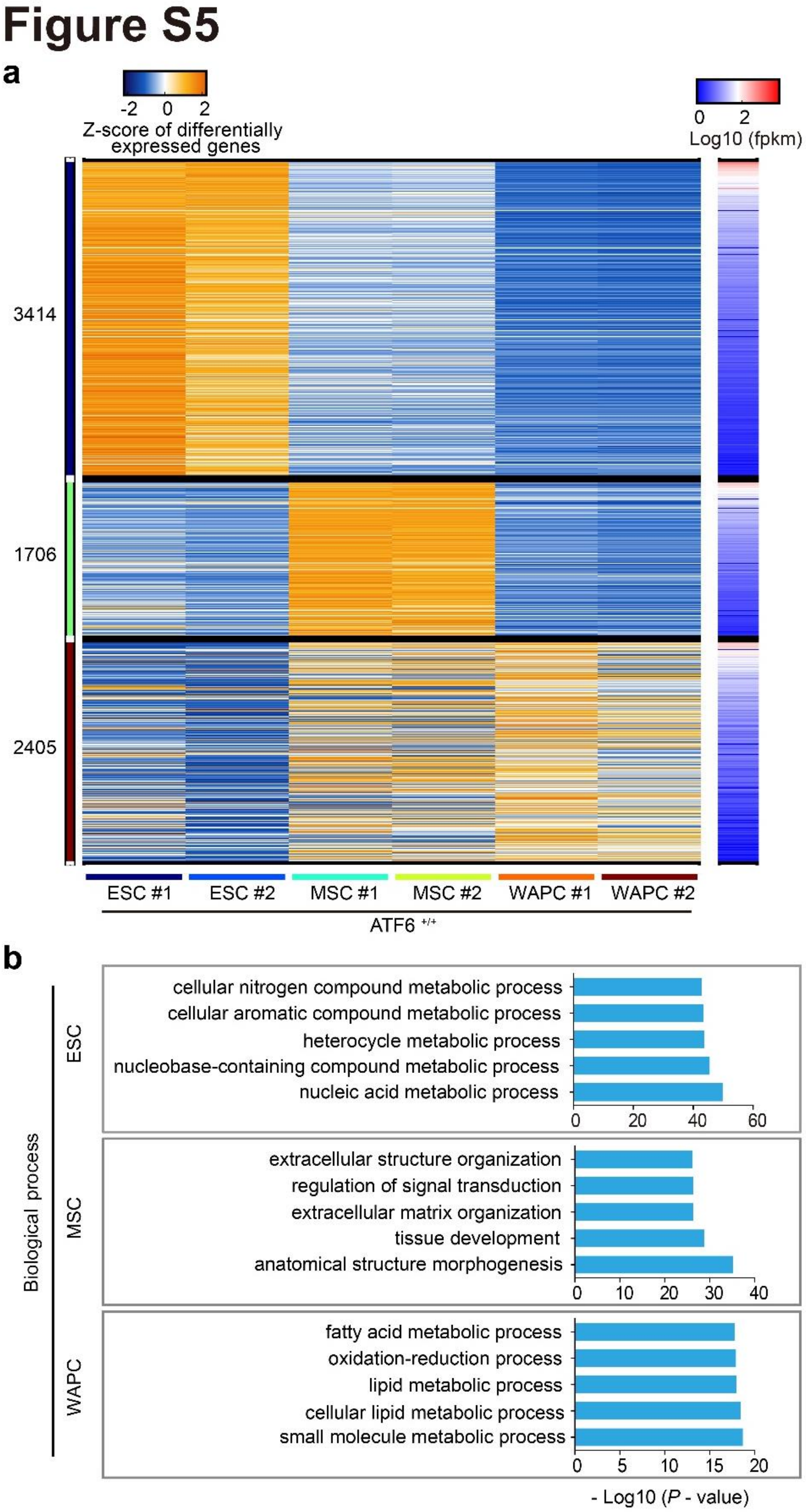

Supplementary Figure S5. Gene expression analyses of wild-type (*ATF6*$^{+/+}$) hESCs, hMSCs and hWAPCs. a A heatmap showing the Z-score normalized expression level of differentially expressed genes (DEG) in *ATF6*$^{+/+}$ hESCs, hMSCs and hWAPCs. **b** Gene ontology (GO) analysis (biological process) showing the top 5 ranked terms enriched in the DEGs of *ATF6*$^{+/+}$ hESCs, hMSCs and hWAPCs (Supplementary Table S9).

Figure S6

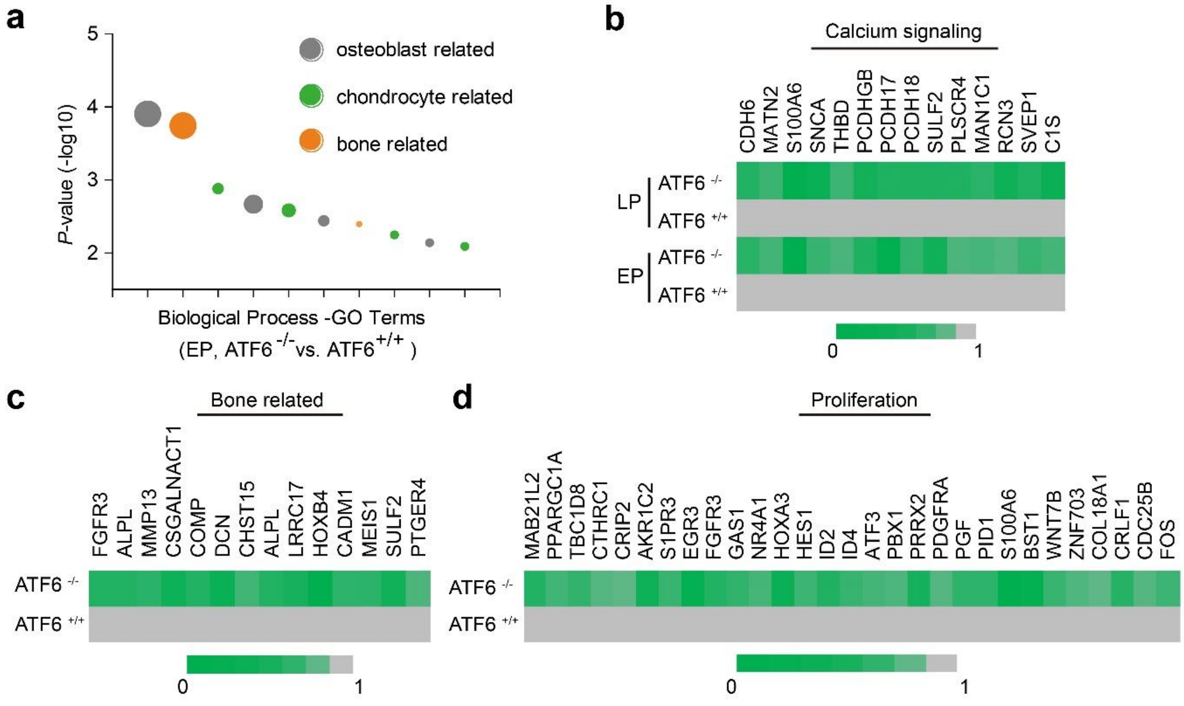

Supplementary Figure S6. Gene expression analyses of WT and ATF6-deficient hMSCs. a Gene ontology (GO) analysis (biological process) of significantly down-regulated genes related to bone development in ATF6-deficient hMSCs. EP, early passage. **b-d** Heatmaps showing the expression pattern of significantly down-regulated genes that are related to calcium signaling (b), bone development (c) and cell proliferation (d) in ATF6-deficient hMSCs.

Figure S7

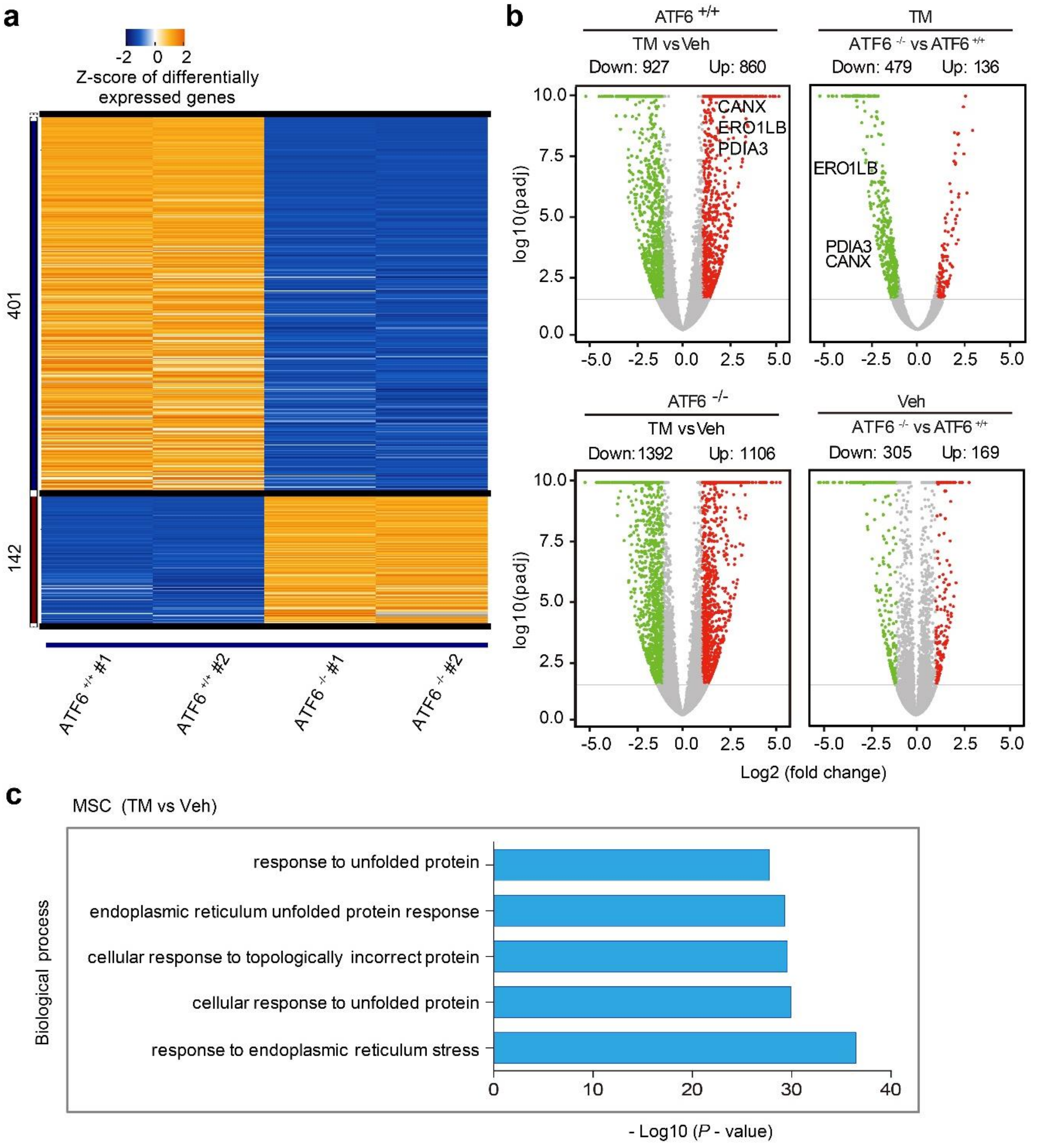

Supplementary Figure S7. Gene expression analyses of WT and ATF6-deficient hESCs, hMSCs and hWAPCs. a A heatmap showing the differentially expressed genes (DEG) upon ATF6 depletion in the late passage hMSCs. **b** Volcano plot showing differentially expressed genes between TM and Veh-treated in the *ATF6*$^{+/+}$ (upper-left) or *ATF6*$^{-/-}$ (lower-left) hMSCs, between *ATF6*$^{-/-}$ and *ATF6*$^{+/+}$ in the presence (upper-left) or absence of TM (lower-left) hMSCs. Representative IARGs were highlighted. Veh, Vehicle, TM, tunicamycin. **c** Gene ontology (GO) analysis (biological process) of significantly upregulated genes in *ATF6*$^{+/+}$ hMSCs upon TM treatment.

Figure S8

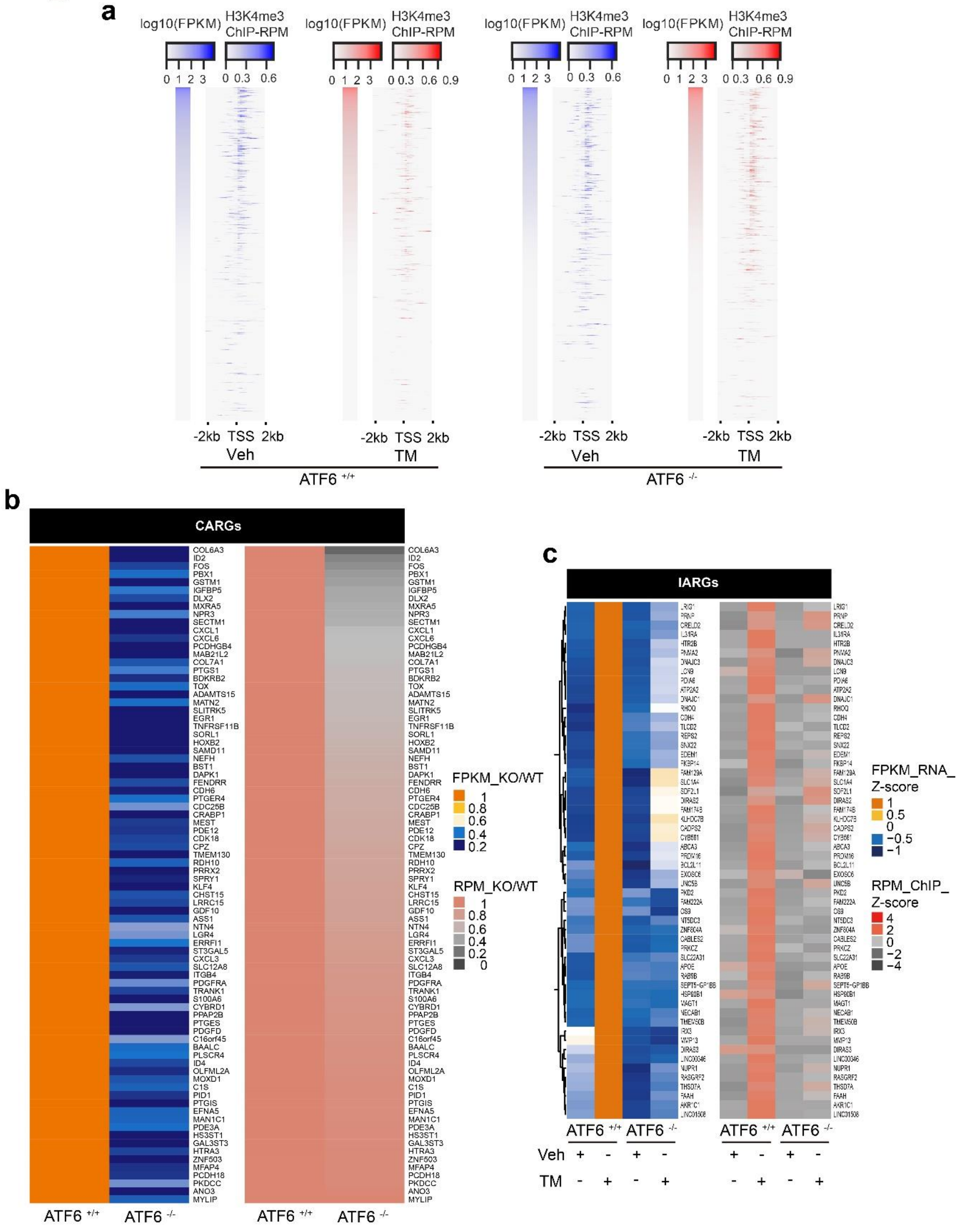

Supplementary Figure S8. H3K4me3 profiles in WT and ATF6-deficient hMSCs. a H3K4me3 signals in the 2 kb region upstream and downstream of transcription start site (TSS) of all genes in WT and ATF6-deficient hMSCs in the presence and absence of TM. And the mRNA expression profile for each sample was also shown on the left. **b-c** Heatmaps showing the gene expression profile (left) and the pattern of the H3K4me3 signal levels (right) of CARGs (b) and IARGs (c).

Figure S9

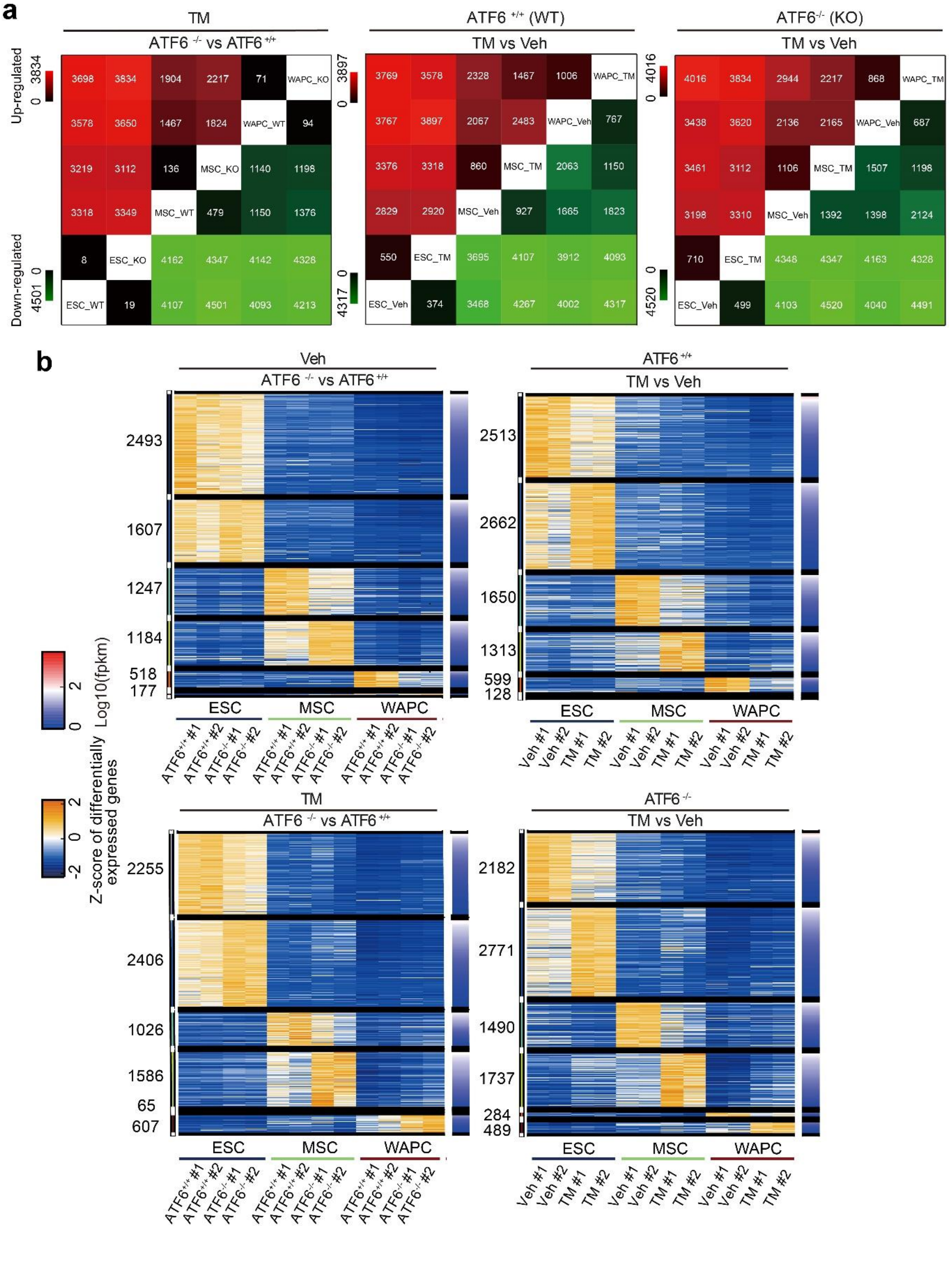
a
TM
ATF6 -/- vs ATF6 +/+
ATF6 +/+ (WT)
TM vs Veh
ATF6-/- (KO)
TM vs Veh
Up-regulated
Down-regulated
b
Veh
ATF6 -/- vs ATF6 +/+
ATF6 +/+
TM vs Veh
TM
ATF6 -/- vs ATF6 +/+
ATF6 -/-
TM vs Veh
ESC
MSC
WAPC
Log10(fpkm)
Z-score of differentially expressed genes

Supplementary Figure S9. Gene expression analyses of WT and ATF6-deficient hESCs, hMSCs and hWAPCs. a Heatmaps showing the number of differentially expressed genes (DEG) between *ATF6*$^{–/–}$ and *ATF6*$^{+/+}$ samples in the presence of TM (left), between TM and Veh-treated in the *ATF6*$^{+/+}$ cells (middle) as well as in the *ATF6*$^{–/–}$ cells (right), cells including hESCs, hMSCs and hWAPCs. WT, wild-type, KO, knockout. **b** Heatmaps showing the Z-score normalization level of differentially expressed genes (DEG) between *ATF6*$^{–/–}$ and *ATF6*$^{+/+}$ samples in the absence (upper-left) or presence of TM (lower-left), between TM and Veh-treated samples in the *ATF6*$^{+/+}$ (upper-right) or *ATF6*$^{–/–}$ samples (lower-right). Genes were first classified as hESCs specific group, hMSCs specific group and hWAPCs specific group, and then divided into two subgroups within these three groups according to its expression level. Veh, Vehicle, TM, tunicamycin.

Figure S10

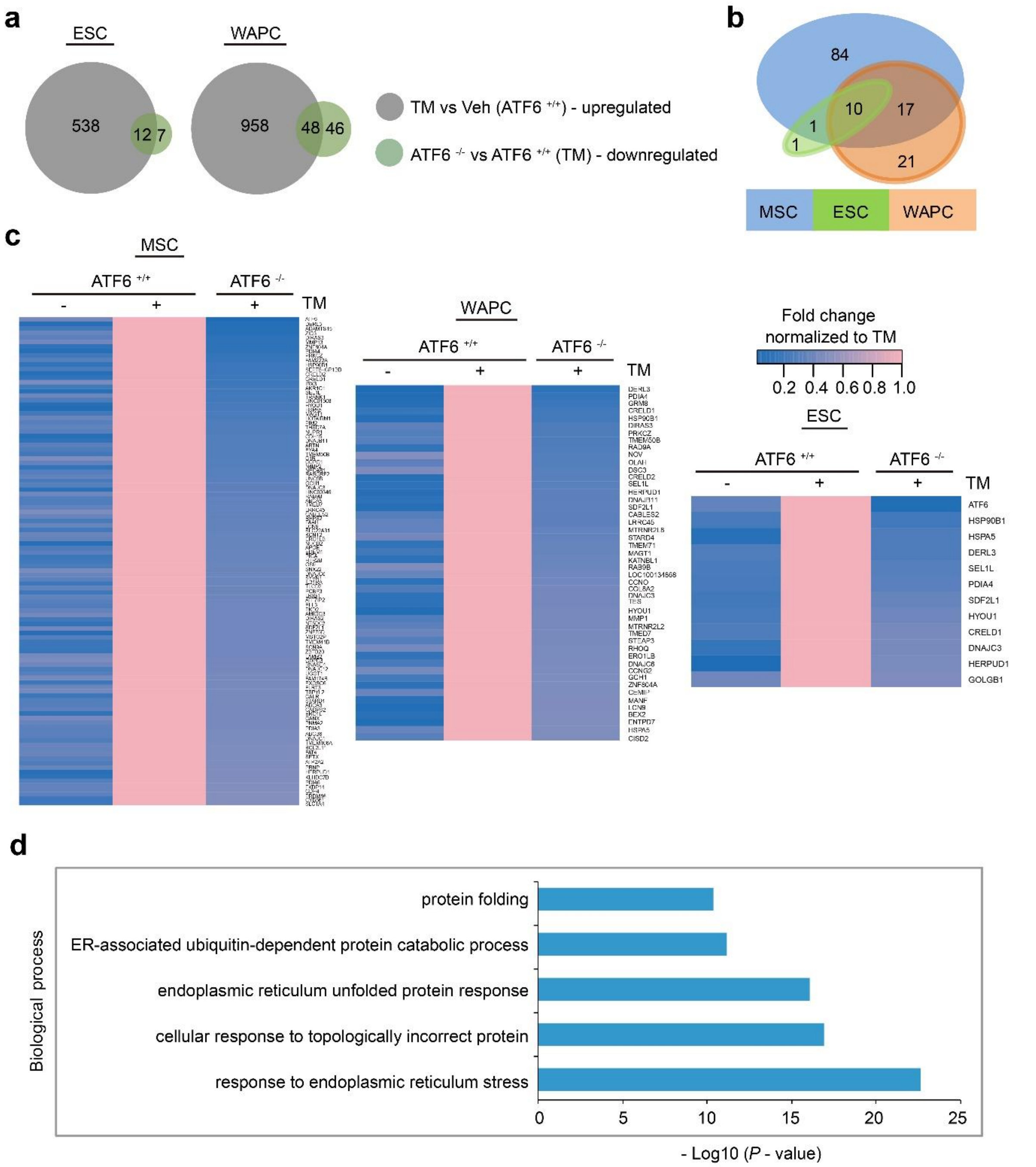

Supplementary Figure S10. Gene expression analyses of IARGs in hESCs, hMSCs and hWAPCs. **a** Venn diagrams showing the number of IARGs in the ESCs and WAPCs. **b** Venn diagrams showing the overlapping IARGs in MSCs, WAPCs and ESCs. **c** Heatmaps showing the expression pattern of IARGs in MSCs, ESCs and WAPCs. **d** Gene ontology (GO) analysis (biological process) showing the enriched GO terms in the IARGs of hMSCs.

Figure S11

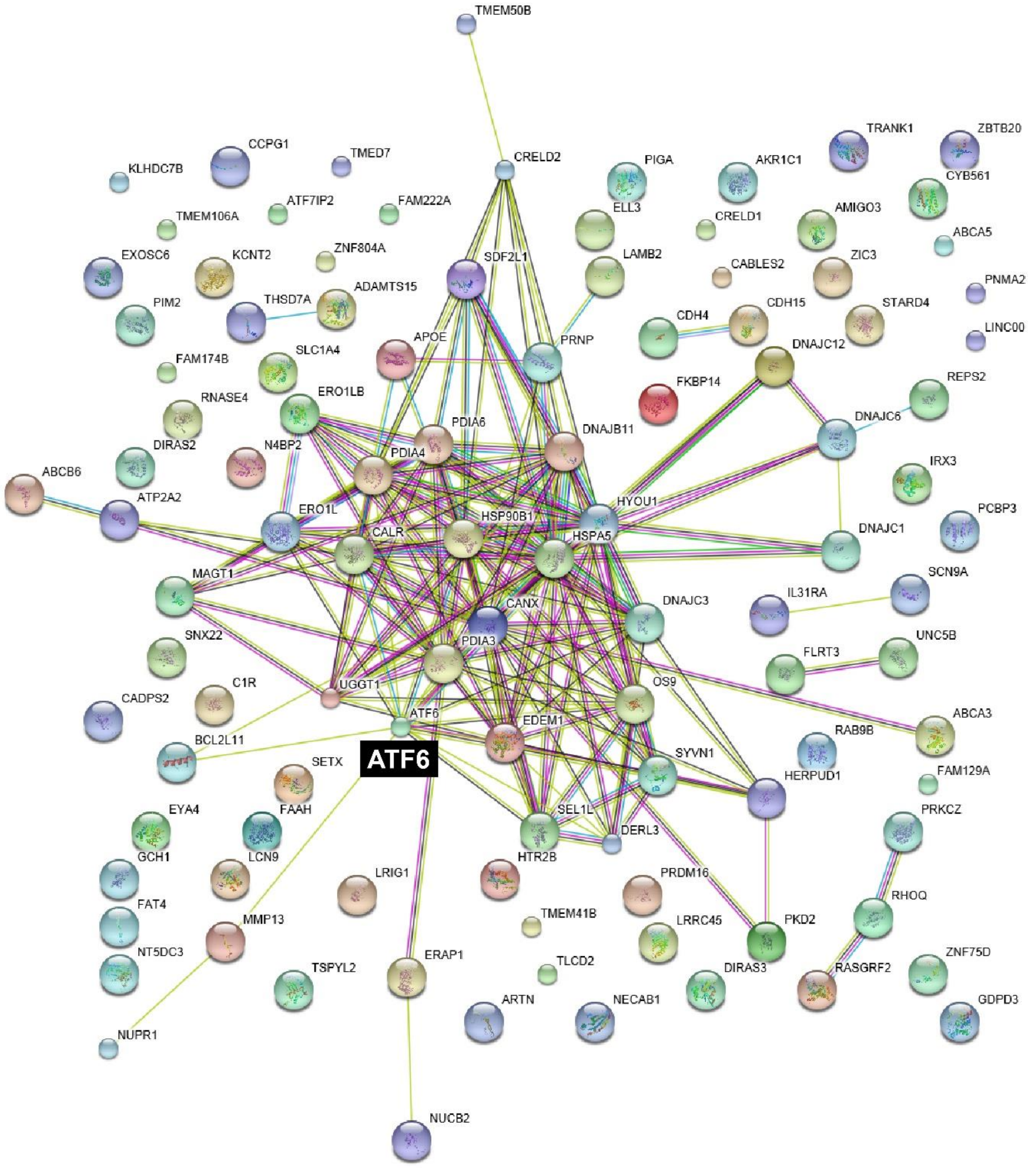

Supplementary Figure S11. Interaction network of IARGs constructed using Search Tool for the Retrieval of Interacting Genes (STRING) database.

112 IARGs in hMSCs were used to construct the interaction network using STRING database, and the resulting chart showed the potential interaction among IARGs. ATF6 was highlighted in the center of the chart.

Figure S12

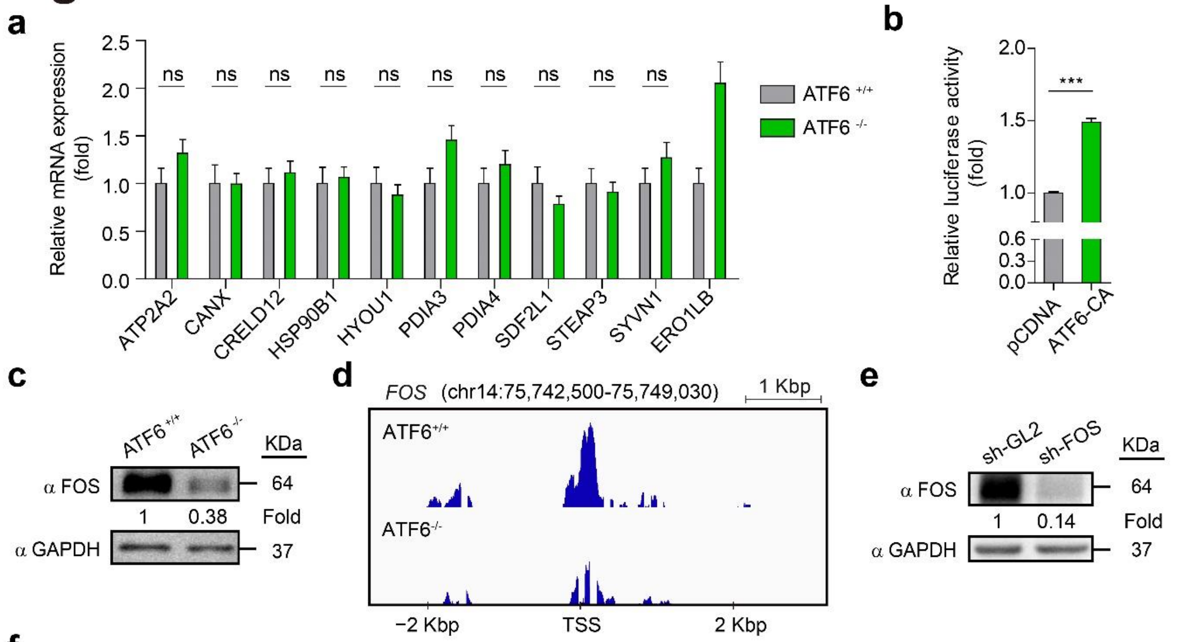

f

	shRNA	Target sequence	Repressing cell proliferation after knockdown
CARGs	FOS	GCGGAGACAGACCAACTAGAAC	+++
	CDC25B	GAGACTGCAGATACCCCTAT	++
	EFNA5	GCCGAGAATATTTCTACAT	+
	DNAJC5	GCTGGCAAGGCTAAGATTA	ns
	S100A6	GCCTTGGCTTTGATCTACA	ns
	LRRC17	GCACATTGAAGAACAACAT	ns
IARGs	CRELD2	CAGAGTATGGATAGCTGCCTTCTCT	++
	ERO1LB	GAAGATGATGGAGAATCATTCTACA	+
	PDIA3	GTATGAAGCTGCAGCTACCAGATTAA	+
	PDIA4	GCTCAGCAAGCGTTCTCCTCCAATT	+
	STEAP3	CAGGAAAGCCAGAACAAGTTCTTCA	ns
	SYVN1	GGCTTTGAGTATGCCATCCTGATGA	ns

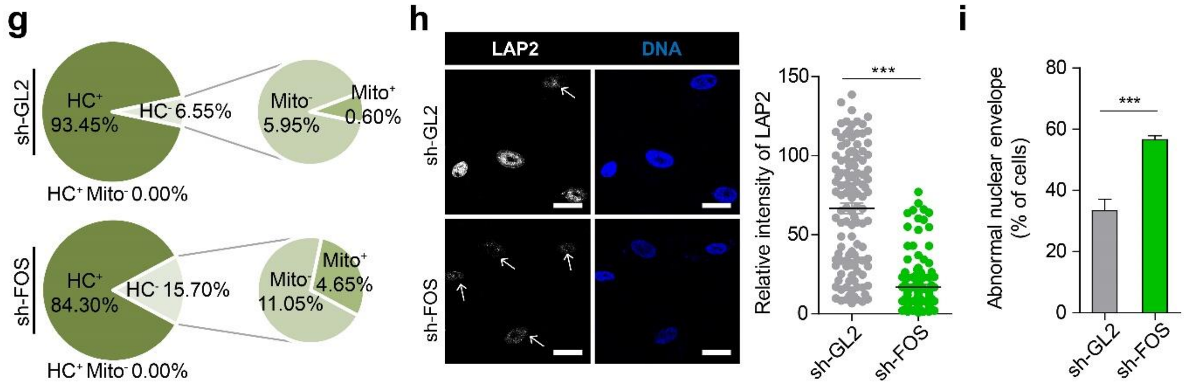

Supplementary Figure S12. Knockdown of *FOS* partially mimics the cellular senescent phenotypes of ATF6- deficient hMSCs. a The mRNA expression changes of several IAGRs in the late passage WT and ATF6-deficient hMSCs. Data were presented as mean ± SEM, n = 3. ns, not significant. **b** The putative ERSE-contained sequence of *FOS* was activated by ATF6-CA as an enhancer. The putative ERSE-contained sequence (~1 kb) of *FOS* was cloned into pGL3-promoter vector, and the luciferase activities were tested and compared in the presence or absence of ATF6-CA. **c** The protein expression of *FOS* in the WT and ATF6-deficient hMSCs. hMSCs were cultured in the basal medium for 20 hrs and then stimulated with culture medium (CM) for 1 hr. Protein was extracted and then Western blotting was performed. GAPDH was used as the loading control. **d** Reduced H3K4me3 signals around transcription start site at the promoter region of *FOS* in the ATF6-deficient hMSCs. **e** Knockdown efficiency of lentiviral sh-FOS vector was evaluated by western blotting. hMSCs transduced with lentiviral sh-GL2 or sh-FOS vectors were cultured in the basal medium for 20 hrs and then stimulated with culture medium (CM) for 1 hr. GAPDH was used as the loading control. **f** A table showing the shRNAs targeting sequences against several CARGs or IARGs, and also the relative repressing cell proliferation ability upon knockdown of indicated genes. **g** Pie charts showing the percentages of cells with reduced heterochromatin and/or abnormal mitochondria observed by TEM in sh-GL2 or sh-FOS transduced hMSCs. “HC” denotes heterochromatin, “HC^{-}” denotes reduced heterochromatin, “Mito” denotes mitochondria, “Mito^{-}” denotes abnormal mitochondria. 168 sh-GL2 cells and 172 sh-FOS cells were imaged and calculated, respectively. **h** Representative immunostaining images showing the LAP2 expression in the sh-GL2 and sh-FOS lentivirus infected hMSCs. Scale bar, 20 μm. The relative intensity of LAP2 in 120 cells was measured with Image J software and the calculated data were shown as mean ± SD, ***P < 0.001. **i** The percentages of cells with abnormal nuclear envelope in sh-GL2 and sh-FOS lentivirus infected hMSCs was calculated and compared, data were shown as mean ± SEM, n = 3, ***P < 0.001.

Supplementary Table S1. Primer sequences used in the study.

Supplementary Table S2. Up- and downregulated genes in *ATF6*$^{-/-}$ hESCs, hWAPCs and early passage hMSCs compared to *ATF6*$^{+/+}$ counterparts.

Supplementary Table S3. Up- and downregulated genes in *ATF6*$^{-/-}$ hMSCs compared to *ATF6*$^{+/+}$ hMSCs (late passage).

Supplementary Table S4. Gene list for IARGs and CARGs.

Supplementary Table S5. GO terms for downregulated genes in *ATF6*$^{-/-}$ hMSCs compared to *ATF6*$^{+/+}$ hMSCs (early passage).

Supplementary Table S6. GO terms for downregulated genes in *ATF6*$^{-/-}$ hMSCs compared to *ATF6*$^{+/+}$ hMSCs (late passage).

Supplementary Table S7. GO terms for upregulated genes in TM-treated WT hMSCs.

Supplementary Table S8. GO terms for IARGs in hMSCs.

Supplementary Table S9. GO terms for cell type-specific expressed genes.

Supplementary Movie 1. Representative movie showing ER architecture in *ATF6*$^{+/+}$ hMSCs.

Supplementary Movie 2. Representative movie showing ER architecture in *ATF6*$^{-/-}$ hMSCs.

Supplementary Movie 3. Representative movie showing ER architecture in mock-transduced *ATF6*$^{+/+}$ hMSCs.

Supplementary Movie 4. Representative movie showing ER architecture in sh-FOS transduced *ATF6*$^{+/+}$ hMSCs.
